# Bromodomain Interactions with Acetylated Histone 4 Peptides in the BRD4 Tandem Domain: Effects on Domain Dynamics and Internal Flexibility

**DOI:** 10.1021/acs.biochem.2c00226

**Published:** 2022-10-10

**Authors:** Sven Wernersson, Romel Bobby, Liz Flavell, Alexander G. Milbradt, Geoffrey A. Holdgate, Kevin J. Embrey, Mikael Akke

**Affiliations:** † Biophysical Chemistry, Center for Molecular Protein Science, Department of Chemistry, Lund University, SE-221 00Lund, Sweden; ‡ Mechanistic and Structural Biology, Discovery Sciences, BioPharmaceuticals R&D, AstraZeneca, CambridgeCB4 0WG, U.K.; § Discovery Biology, Discovery Sciences, BioPharmaceuticals R&D, AstraZeneca, Cambridge Science Park, CambridgeCB4 0WG, U.K.

## Abstract

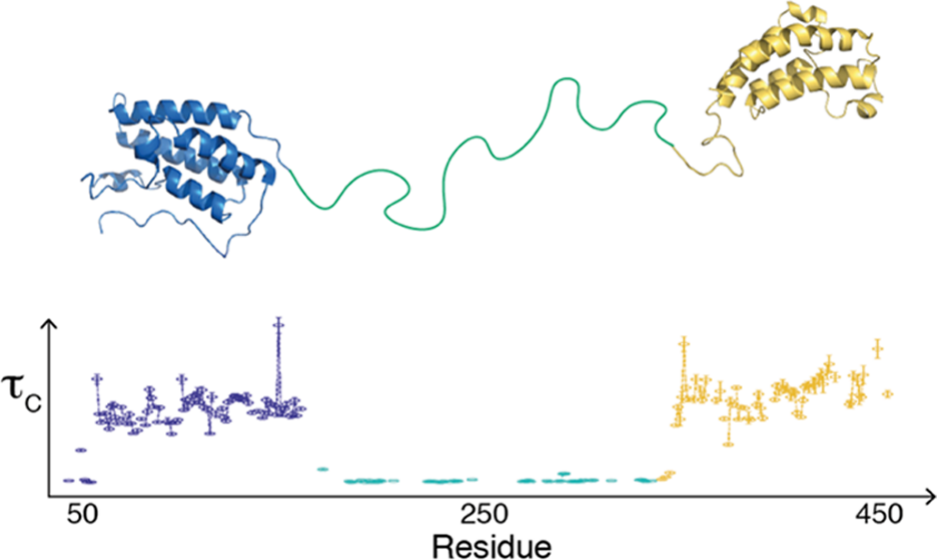

The bromodomain and extra-terminal (BET) protein BRD4 regulates gene expression via recruitment of transcriptional regulatory complexes to acetylated chromatin. Like other BET proteins, BRD4 contains two bromodomains, BD1 and BD2, that can interact cooperatively with target proteins and designed ligands, with important implications for drug discovery. Here, we used nuclear magnetic resonance (NMR) spectroscopy to study the dynamics and interactions of the isolated bromodomains, as well as the tandem construct including both domains and the intervening linker, and investigated the effects of binding a tetra-acetylated peptide corresponding to the tail of histone 4. The peptide affinity is lower for both domains in the tandem construct than for the isolated domains. Using ^15^N spin relaxation, we determined the global rotational correlation times and residue-specific order parameters for BD1 and BD2. Isolated BD1 is monomeric in the apo state but apparently dimerizes upon binding the tetra-acetylated peptide. Isolated BD2 partially dimerizes in both the apo and peptide-bound states. The backbone order parameters reveal marked differences between BD1 and BD2, primarily in the acetyl-lysine binding site where the ZA loop is more flexible in BD2. Peptide binding reduces the order parameters of the ZA loop in BD1 and the ZA and BC loops in BD2. The AB loop, located distally from the binding site, shows variable dynamics that reflect the different dimerization propensities of the domains. These results provide a basis for understanding target recognition by BRD4.

## Introduction

Epigenetic regulation of gene expression involves switching between chromatin conformations that are either compact, in which gene expression is silenced, or open, in which the transcriptional machinery can access DNA. Post-translational modification of histones constitutes an important determinant of such regulation that responds to physiological and environmental signals. Epigenetic “writer” and “eraser” enzymes introduce and remove, respectively, post-translational modifications of histones, while “reader” domains recognize the modifications and aid in initiating transcription through various modes of action.^1^ Acetylation of lysine side chains on histone tails is a central example of post-translational modification that is recognized by the “reader” bromodomain (BD).^2^ Among the many BDs present in the human genome, the BDs of the bromodomain and extra-terminal (BET) family of proteins have emerged as an important class of transcriptional coactivators involved in cell cycle progression, transcriptional activation, and elongation. In particular, the BET protein BRD4 can bind not only to acetylated histones but also directly to various transcription factors in an acetylation-dependent manner.^3,4^ BRD4 is involved in the transcription of oncogenes and pro-inflammatory cytokines and chemokines, making it an important target for the treatment of several diseases, including inflammation and cancer,^5−7^ using small-molecule inhibitors of BDs.^4^

**Figure 1:**
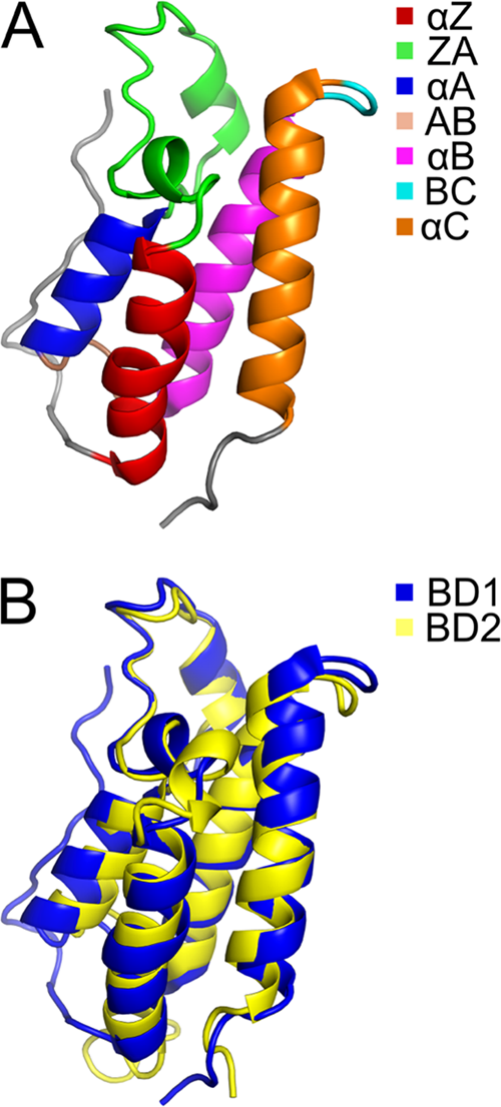
BRD4 bromodomain structures. (A) Overview of bromodomain structure, exemplified here by BD1 (PDB-ID: 4CLB(14)), with the different secondary structure elements highlighted by color. The binding site is located at the top of the structure in this view. (B) Ribbon representation of superimposed structures of BD1 (blue, PDB-ID: 4CLB(14)) and BD2 (yellow, PDB-ID: 2LSP(15)). Bound ligands are not shown. The figure was prepared using PyMOL.^16^

BET proteins contain two 110-residue-long BDs, denoted BD1 and BD2, in addition to the extra-terminal domain, which is located C-terminally of the BDs. The domains are separated by long unstructured segments; in BRD4, the segment between BD1 and BD2 is roughly 180 residues long. The BET BDs show preference for binding diacetylated peptides with the acetylated lysine (Kac) residues close in sequence.^4^ The BD structure is an antiparallel bundle of four α-helices (α_Z_, α_A_, α_B_, and α_C_), where the two interhelical loops (ZA and BC) at one end of the molecule form a hydrophobic pocket that binds the Kac-containing peptide motifs (Figure 1). Crystal structures have revealed the detailed interactions between BD residues and the Kac peptides.^4^ The ZA loop, comprising 11 residues (85–95) in BD1 and 16 residues (373–388) in BD2, is considerably longer and known to be much more flexible than the BC loop, comprising five residues in both BD1 (140–144) and BD2 (433–437). Molecular dynamics (MD) simulations have indicated that the dynamics of residues in the BC and ZA loops lead to switching between occluded and open binding sites that are important for binding.^6,8−11^ These and other observations have led to the concept that dynamics, rather than structure, is key to achieving inhibitor selectivity between BD1 and BD2.^8,12,13^

The mechanistic significance of the tandem arrangement of BDs has not been resolved fully, but it appears that BD1 alone is sufficient to bind BET proteins to chromatin and maintain steady-state gene expression, whereas both BD1 and BD2 are required to achieve rapid increase in gene expression in response to inflammatory signals.^7^ While chromatin binding is known to involve each BD individually,^12,17^ tandem BDs have been implicated in binding multiple acetyl-lysine-containing targets at different points during transcription in a coordinated way,^18,19^ and BD-mediated dimerization of BRD4 on chromatin has been detected in vivo.^20^ Bivalent inhibitors of BET bromodomains bind simultaneously to BD1 and BD2,^19,21,22^ demonstrating conformational flexibility of the intermediate linker region connecting the two domains. These observations indicate important functional roles of dimerization and flexibility of the tandem BD arrangement, but little is currently known about the extent of interdomain flexibility, interdomain interactions, or the dynamic consequences of binding Kac peptides corresponding to histone tails.^23^ To reach a complete understanding of the role of dynamics in BRD4 function, it is thus critical to address not only the internal dynamics of individual BDs but also the dynamics of the intact tandem BDs, including the relative orientational dynamics of the BDs and the dynamics of interdomain segment.

Here, we report a comparative analysis of the ligand-binding properties and conformational dynamics of the tandem BDs from BRD4, as well as the isolated domains BD1 and BD2. Using nuclear magnetic resonance (NMR) spectroscopy, we measure domain-specific affinities for a tetra-acetylated H4 histone peptide in the context of the tandem arrangement and characterize the conformational dynamics of the BDs, as well as the linker region between them. Rotational diffusion correlation times reveal differences between BD1 and BD2 in their propensities to form dimers. Our results show that tetra-acetylated H4 histone peptides bind to the individual domains, rather than forming bivalent complexes involving both BD1 and BD2. The results pinpoint differences between the two BDs in their conformational dynamics on both fast (picosecond to nanosecond) and slow (microsecond to millisecond) timescales, most notably involving the ZA and BC loops. Furthermore, the two domains respond differently to binding Kac peptides.

## Materials and Methods

### Protein Expression and Purification

BRD4 (Uniprot accession number O60885) genes were cloned into pET28 expression vectors containing an N-terminal His_6_-tag followed by a tobacco etch virus (TEV) protease site. Sequences for the individual constructs covered residues N44 to E168 for the N-terminal bromodomain, BD1; H341 to E460 for the C-terminal bromodomain, BD2; and N44 to E460 for the tandem BRD4(1,2). The TEV protease-digested BD1 and BRD4(1,2) constructs retained four non-native residues (G40, S41, H42, M43) prior to the native N44, whereas the BD2 construct retained a non-native Gly–Gly sequence prior to its native H341. Expression and purification closely followed the protocol described previously.^21^ Uniform labeling with ^15^N and ^13^C isotopes was achieved by expression in minimal M9 medium with ^15^NH_4_Cl (Sigma-Aldrich) and ^13^C glucose (Cambridge Isotope Laboratories) as the sole sources of nitrogen and carbon, respectively. In addition, ^15^N and ^13^C labeled Celtone medium (Cambridge Isotope Laboratories) was supplemented to the growth medium at 5 g/L. For perdeuteration, either glucose-*d*_7_ for U-[^2^H,^15^N] labeling or ^13^C-glucose-*d*_7_ for [^2^H,^13^C,^15^N] labeling was used in an M9/D_2_O medium with supplements of ^2^H variants of the Celtone Base Powder.

### Size Exclusion Chromatography (SEC)

Size exclusion chromatography was performed using Superdex 75 resin in a 3.2/300 column (Cytiva). The buffer contained 20 mM 4-(2-hydroxyethyl)piperazine-1-ethanesulfonic acid (HEPES) pH 7.4, 100 mM NaCl, and 1 mM tris(2-carboxyethyl)phosphine (TCEP). Samples were loaded as either 50 μL of 135 μM or 20 μL of 350 μM protein solution. Retention times were calibrated using 10 μL of Bio-Rad gel filtration standards.

### NMR Sample Preparation

The NMR samples contained 135 μM protein, i.e., BD1, BD2, or BD4(1,2), dissolved in a buffer comprising 20 mM Na_2_HPO_4_, 1 mM TCEP, and 7/93% D_2_O/H_2_O at pH 6.8. Two additional samples were prepared to monitor concentration-dependent chemical shift changes in BD2. These samples contained 120 or 526 μM BD2, dissolved in said buffer. Peptide-bound samples contained in addition 0.90 mM H4Kac4 in the case of BD1 and BD2, or 1.5 mM H4Kac4 in the case of BRD4(1,2). The resulting saturation levels are 99% (isolated BD1), 91% (isolated BD2), 98% (BD1 in the tandem construct), and 92% (BD2 in the tandem construct).

### NMR Spectroscopy

All NMR experiments were performed at 30 °C on Bruker AV 600 and AVIII 800 spectrometers equipped with 5 mm *z*-gradient ^1^H/^13^C/^15^N TCI cryoprobes. Temperature calibration was performed with a 99.8% methanol-*d*_4_ sample.^24^ Proton chemical shifts were referenced to 4,4-dimethyl-4-silapentane-1-sulfonic acid (DSS), whereas ^15^N and ^13^C chemical shifts were indirectly referenced as described.^25^ The assignment strategy for the backbone ^1^H^N^, ^15^N, ^13^C′, ^13^C^α^, and side-chain ^13^C^β^ chemical shifts utilized standard triple-resonance experiments.^26^ Backbone assignments for isolated BD1 were obtained using ^1^H–^15^N SOFAST heteronuclear multiple-quantum coherence (HMQC),^27,28^ 3D CBCANH,^29^ CBCA(CO)NH,^30^ HN(CO)CA,^31^ HNCA,^32^ and HNCO^32^ experiments. All subsequent triple-resonance experiments contained the transverse relaxation optimized spectroscopy (TROSY)-based detection scheme.^33^ Backbone assignments for isolated BD2 are available from BMRB accession numbers 15057, 18439, and 19738. The BD2 assignments were manually confirmed using a 3D HNCACB experiment. The backbone assignments for the tandem bromodomain construct BRD4(1,2) were obtained using three-dimensional HNCACB, HN(CO)CACB, HNCA, HN(CO)CA, HNCO, HNCACO, (H)N(COCO)NH, and (HN)CO(CO)NH experiments.^34^ All multidimensional experiments were acquired using a nonuniform sampling scheme with Poisson gap distribution as described.^35^ Spectra were processed with NMRPipe^36^ and analyzed with CcpNmr analysis.^37^ Nonuniformly sampled spectra were reconstructed using the istHMS method.^35^ Figures containing NMR spectra were plotted using the Python-based program nmrglue.^38^

### NMR ^15^N Relaxation Experiments

TROSY-based ^15^N relaxation experiments^39^ were performed at static magnetic field strengths of 14.1 and 18.8 T. ^15^N *R*_1_ relaxation experiments were acquired with delays of 100, 200, 300, 400, 500, 600, 800, 1000, 1300, 1600, 1900, and 2200 ms at 18.8 T, and 100, 200, 300, 400, 500, 600, 800, 1200, 1500, 1600, and 2000 ms at 14.1 T. ^15^N *R*_2_ relaxation experiments were acquired with delays of 15.7, 31.4, 47.0, 62.7, 78.4, 94.1, 125.4, 141.1, 156.8, 188.2, and 203.8 ms at both 14.1 and 18.8 T. Steady-state {^1^H}–^15^N-heteronuclear nuclear Overhauser enhancement (NOE) data were measured from pairs of interleaved spectra recorded with or without ^1^H saturation during the 7.0 s recycle delay, denoted NOE and control, respectively. ^1^H saturation was applied as a train of high-power 120° pulses.^40^ Transverse cross-correlation relaxation rate constants (η*_xy_*) were measured as the difference in the relaxation rates of the TROSY and anti-TROSY components of the NH doublet,^41,42^ using relaxation delays of 2, 4, 6, 15, 25, 40, 50 ms with duplicate data acquired at 15 and 25 ms. ^15^N *R*_1_, *R*_2_ and η_*xy*_ relaxation rate constants were obtained by nonlinear least-squares fitting of peak intensities at measured relaxation delays, as implemented in the program relax,^43^ whereas the steady-state {^1^H}–^15^N NOE values were calculated from peak intensity ratios obtained from spectra acquired in the presence and absence of proton saturation, with uncertainties in peak intensities estimated from the baseplane noise. Uncertainties of the relaxation rates were obtained using duplicate delays and standard errors were estimated from a sample of 500 Monte Carlo simulations of the uncertainties for each dataset.^44^ Trimmed averages were calculated in MATLAB by first calculating the mean and standard deviation of the full dataset. Data points outside of one standard deviation from the mean were then removed, and a new mean was calculated from the remaining data points.

### Model-Free (MF) Relaxation Data Analysis

The extended model-free formalism^45−48^ was used to analyze the ^15^N relaxation data for BRD4 with the method for the combined optimization of the global diffusion tensor and local model-free parameters implemented in the relax program (version 3.3.6).^49^ The analysis assumed an N–H bond length (*r*_NH_) of 1.02 Å and a chemical shift anisotropy (CSA) (Δσ_CSA_) of −172 ppm. The N–H bond vector orientations were extracted from the BD1 X-ray and BD2 NMR-derived structures, PDB entries 4CLB(14) and 2LSP,^15^ respectively. The ligands in the PDB structures were removed prior to analysis. All residues included in the analysis were represented by relaxation data recorded at both field strengths.

### Diffusion Tensor Analysis

The MATLAB-based version of the rotdif program^50^ was modified for use with η*_xy_* transverse cross-correlated relaxation rates to estimate the overall rotational diffusion tensor. This approach avoids potential problems caused by exchange contributions to the transverse relaxation rate, which can occur in *R*_2_ but not in η*_xy_*. The ratio of the spectral densities *J*(ω_N_) and *J*(0) is calculated as
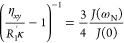
where
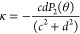
and *d* is the dipolar coupling constant (μ_0_*h*γ_H_γ_N_)/(8π^2^*r*_NH_^3^), μ_0_ is the permeability of free space, *h* is Planck’s constant, γ_H_ and γ_N_ are the gyromagnetic ratios of hydrogen and nitrogen, respectively, *r*_NH_ is the N–H bond length (1.02 Å), *c* is the CSA coupling constant (ω_N_Δσ_CSA_)/3 with Δσ_CSA_ = −172 ± 20 ppm, *P*_2_(θ) is the second order Legendre polynomial, and θ = 15 ± 10° is the angle between the N–H internuclear vector and the unique axis of the chemical shielding tensor. The values of Δσ_CSA_ and θ correspond to conservative averages taken from the literature.^51−53^*R*_1_′ is the longitudinal relaxation corrected for high-frequency spectral density:^54^
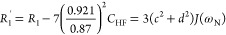
where the correction factor (*C*_HF_) for the high-frequency components is calculated under the assumption that *J*(ω) ∝ ω^–2^ at ω ≈ ω_H_, yielding
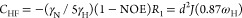


The HydroNMR software^55,56^ was used to calculate diffusion tensors and correlation times for the isolated BD1 and BD2 domains, based on PDB structures 4CLB(14) and 2LSP,^15^ respectively. The ligands in the PDB structures were removed prior to analysis. The temperature was set to 303 K and the solvent viscosity to 798·10^–6^ Pa s. An effective atomic radius of 3 Å was used in accordance with Halle & Davidovic.^57^ The three principal values of the diffusion tensor are defined as *D*_xx_, *D*_yy_, and *D*_zz_, with *D*_zz_ ≥ *D*_yy_ ≥ *D*_xx_. Further, the rotational diffusion correlation time (τ_c_) and diffusion anisotropy (*D*_∥_/*D*_⊥_) are obtained from the relationships:
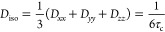

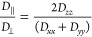


### Spectral Density Mapping

Spectral density mapping^58^ was performed using data obtained at 14.1 and 18.8 T with approximate expressions for *J*(0.921ω_H_) and *J*(0.955ω_H_) obtained by extrapolation from the static magnetic field dependence of the relaxation data using a first-order Taylor series expansion of *J(*ω) at the 0.870ω_H_ frequency^58^
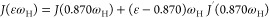
where ε = 0.921 or 0.955, and *J*′(0.870ω_H_) is the first derivative of *J*(ω) at the 0.870ω_H_ frequency, estimated from the difference in *J*(0.921ω_H_) between the different static magnetic field strengths. The spectral density mapping calculations used Δσ = −173 ± 7 ppm and *r*_NH_ = 1.04 Å.^51^

Exchange contributions (*R*_ex_) to *R*_2_ were estimated by comparing auto- and cross-correlated relaxation rates, following published protocols.^59^ In this approach, *R*_ex_ can be estimated at a specified static magnetic field strength, (*B*_0_) as:
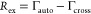


Γ_auto_ and Γ_cross_ were calculated from *R*_1_, *R*_2_, *NOE*, and η*_xy_* data acquired at 18.8 T:
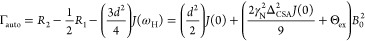

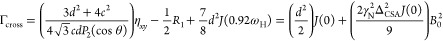
where the spectral densities *J*(ω_H_) and *J*(0.92ω_H_) were obtained by spectral density mapping as described above, using θ = 19.6° ± 2.5°,^51^ and Θ_ex_ = *R*_ex_/*B*_0_^2^. This analysis is sensitive to errors arising from site-specific variations in CSA.

Errors were propagated using Monte Carlo simulations.^44^ For each residue, 10,000 log-normal distributed points were randomly generated for each variable (*R*__1__, *R*__2__, *NOE*, η*_xy_*, Δσ_CSA_, etc.) using a standard deviation equal to the error estimated from the fitted relaxation data.

### *K*_d_ Determination

A peptide mimicking a tetra-acetylated histone 4 (H4) tail, comprising residues 1–16 of H4 with Nε-acetylation at K5, K8, K12, and K16 (denoted H4Kac4) was purchased from Cambridge Research Biochemicals (Cambridge, UK). The peptide was dissolved at a concentration of 10 mM in 0.2 M Na_2_HPO_4_ pH 6.8. For binding titrations, all NMR samples contained 0.065 mM of U-[^2^H,^15^N]-labeled BRD4(1,2) or U-[^13^C,^15^N]-labeled BD1/BD2. The ^1^H and ^15^N chemical shift changes were followed by collecting ^1^H–^15^N TROSY experiments at six ligand concentrations: (30, 60, 119, 235, 461, and 889) μM. The combined chemical shift perturbation was calculated using the equation
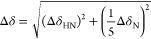
where ΔδHN and ΔδN denote the chemical shift differences between the peptide-bound and apo states for ^1^H^N^ and ^15^N, respectively.

We measured the dissociation constants for the interaction of BRD4 BDs with different ligands by monitoring the chemical shift changes of BRD4 BDs from the apo to the peptide-bound form during titration. When the exchange rate is greater than the chemical shift difference between the free and bound states, the observed chemical shift perturbation at each titration point (Δδ_obs_) is the population-weighted average between the chemical shifts of the free and bound states obtained by the following mass action binding isotherm equation for binding to a single site (valid for BD1 and BD2):
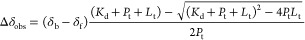
where δ_b_ and δ_f_ are the chemical shifts of the bound and free states, respectively; *P*_t_ and *L*_t_ are the total concentrations of protein and ligand, respectively, at each titration point; and *K*_d_ is the dissociation constant. In the case of BRD4(1,2), the chemical shift perturbations were analyzed using the corresponding coupled equations valid for simultaneous binding to two sites (1 and 2):
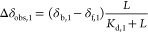

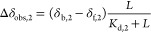
where *L* is the concentration of free ligand, *L* = *L*_t_ – *P*_1_*L* – *P*_2_*L*, and *P*_i_*L* indicates the concentration of protein–ligand complex with the ligand bound to site i, which is obtained as
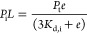
where
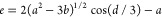

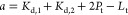

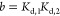

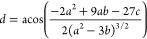

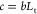


We performed global, nonlinear fits of the above equations to the experimental titration data using the Levenberg–Marquardt algorithm implemented in the GraFit package version 6.0.5 (Erithacus Software Ltd., Staines). Estimated errors are reported as 1 standard deviation based on the covariance matrix.

## Results and Discussion

We used NMR spectroscopy to investigate ligand interactions, intramolecular dynamics, and rotational diffusion of the BRD4 bromodomains. NMR makes it possible to determine the domain-specific binding affinities in the context of the tandem construct. We compared the dynamics of the isolated domains and the tandem construct, in both the free and ligand-bound states. Furthermore, we characterized the dynamics of the disordered interdomain linker segment and its effects on the structure, dynamics, and interactions of the BDs. NMR provides an opportunity to study all of these properties under identical sample conditions; this contrasts with many previous studies of BET bromodomains, which have involved multiple methods involving different conditions to investigate the interactions of BD1 and BD2. By studying ligand binding and the rotational diffusion properties under identical conditions, we arrive at a consistent model for the coupled changes in structure, dynamics, and interactions upon ligand binding to BRD4 that resolves partly conflicting interpretations of previously reported results.

### Chemical Shift Differences between the Isolated and Tandem Domains Reveal Differences in Domain Interactions

**Figure 2:**
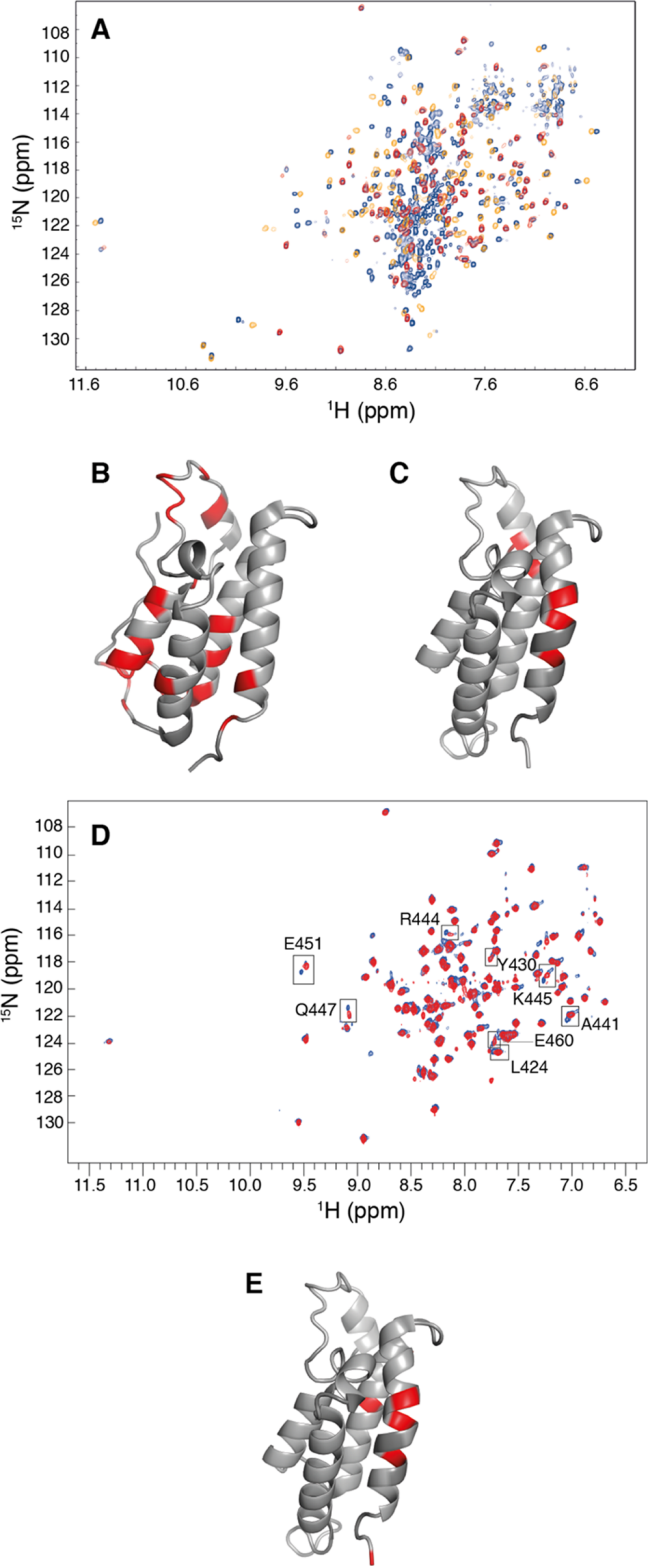
^1^H–^15^N TROSY spectra of the tandem BRD4-construct and the two isolated domains BD1 and BD2 and chemical shift differences between the isolated and tandem constructs. (A) Superimposed ^1^H–^15^N TROSY spectra for the tandem construct BRD4(1,2) (blue), isolated BD1 (yellow), and isolated BD2 (red), (B, C) Absolute backbone amide chemical shift differences (Δδ) between tandem and isolated constructs mapped onto the structures of (B) BD1, PDB-ID 4CLB,^14^ and (C) BD2, PDB-ID 2LSP.^15^ Residues with Δδ > 0.1 are colored red. (D) Overlay of spectra acquired on isolated BD2 at concentrations of 120 μM (red) and 526 μM (blue). Boxes indicate peaks showing significant chemical shift changes and two sets of peaks at the higher concentration. (E) Residues with Δδ > 0.05 between 526 and 120 μM mapped onto the structure of BD2. The chemical shift differences are plotted versus residue number in Figure S1. All spectra were acquired at a static magnetic field strength of 18.8 T. Panels (B), (C), and (E) were prepared using PyMOL.^16^

To enable residue-specific studies of BD interactions and dynamics, we previously reported the use of segmental labeling of BRD4(1,2) to assign the backbone amide ^1^H and ^15^N resonances of BD1 and BD2 in the tandem domain construct, BRD4(1,2), together with the interdomain linker.^60^ Here we compare the chemical shifts of the isolated domains with those recorded for the tandem construct. Resonance assignments were aided by previously reported chemical shift datasets of the BRD4 bromodomains, e.g., Biological Magnetic Resonance Bank entries 50145 and 50146.^61^ Standard triple-resonance experiments (see the Materials and Methods section) were used to obtain backbone assignments of the isolated domains BD1 and BD2 at a level of 98 and 93%, respectively, for nonproline residues. For BRD4(1,2), standard triple-resonance experiments performed on both uniformly and segmentally labeled protein^21,60^ enabled nearly complete assignments of BD1 (88%) and BD2 (87%). However, resonance assignment of the interdomain linker region in BRD4(1,2) was nontrivial, due to severe resonance overlap in this region (T169–S348), which has properties characteristic of an intrinsically disordered protein region, including an abundance of repeat sequences and a high proportion of proline residues. Using special triple-resonance experiments designed to establish sequential connections across proline residues and stretches with high chemical shift degeneracy,^34^ we succeeded to assign the interdomain linker region to a completeness of 69%. In summary, despite a lower overall completeness of assignments in BRD4(1,2), a high percentage of backbone assignments were obtained for the two bromodomains. As might be expected, the 2D ^1^H–^15^N TROSY spectra of the tandem domain BRD4(1,2) show a high degree of resonance overlap with most of the resonances from the linker region having a narrow chemical shift dispersion in the ^1^H dimension reflecting its largely unstructured nature (Figure 2A). In addition, there is significant variation in the peak intensities, indicating nonuniform dynamics in BRD4(1,2).

The NMR spectra reveal chemical shift differences between BD1 in the isolated and tandem constructs. The overall chemical shift difference is 0.10 ppm with greater differences (Δδ = 0.37–0.45 ppm) observed for residues N54, K55, Y118, W120, N121, and A122. These residues are all located adjacent to the C-terminus of BD1 and residues 118–122 are situated in the AB loop, showing that the linker affects this region of BD1 in the tandem construct (Figure 2B). In the case of BD2, the chemical shift differences are much smaller with an average of 0.03 ppm, but still indicate perturbations of Δδ = [(Δδ_HN_)^2^ + (0.2Δδ_N_)^2^]^1/2^ = 0.11–0.17 ppm in primarily three regions around residues I394, N428, K445, Q447, and E451. These residues are all located on the same side of the helix bundle (Figure 2C). Interestingly, this region has previously been implicated in homo-dimerization based on homology with the BD1 domain of BRD2, which is known to form homodimers.^62,63^ Thus, the differences in chemical shifts between the isolated domain and tandem construct might indicate that the presence of the linker and the other domain affects the tendency of BD2 to form homodimers. To investigate whether isolated BD2 dimerizes we acquired ^1^H–^15^N correlation spectra as a function of BD2 concentration. Figure 2D shows an overlay of spectra acquired on samples containing 120 and 526 μM BD2, which reveals chemical shift changes for a subset of residues. L424, Y430, K445, Q447, E451, and E460 all show Δδ > 0.05 ppm, while R444 and A441 also show significant changes (primarily in the ^1^H dimension), albeit with Δδ < 0.05 ppm. The residues with larger shift changes actually give rise to two separate peaks at the higher concentration (e.g., K445, Q447, E451), indicating that they are in slow exchange on the chemical shift timescale. The residues showing Δδ > 0.05 ppm define a region on the structure (Figure 2E) that agrees well with that highlighted in Figure 2C. These results provide a strong indication that isolated BD2 indeed forms transient homodimers. Thus, BD2 is undergoing slow-to-intermediate exchange between monomeric and dimeric states with a dissociation rate on the order of 100 s^–1^. The relative populations of BD2 in the monomeric and dimeric states can be estimated from the average relative peak intensities of the two sets of peaks in the spectrum at 526 μM: *p*_d_ = 1 – *p*_m_ = 0.57 ± 0.05, which in turn yield a dimer dissociation constant of 350 ± 90 μM, calculated as *K*_d_ = 2*p*_m_^2^*P*_t_/*p*_d_ (where the factor 2 enters because *p*_d_ refers to the population of BD2 molecules in the dimer, rather than the population of dimers per se). Below, we further address the issue of BD2 dimerization using relaxation measurements.

### Binding of Tetra-Acetylated H4 Peptide to BD1 and BD2

**Figure 3:**
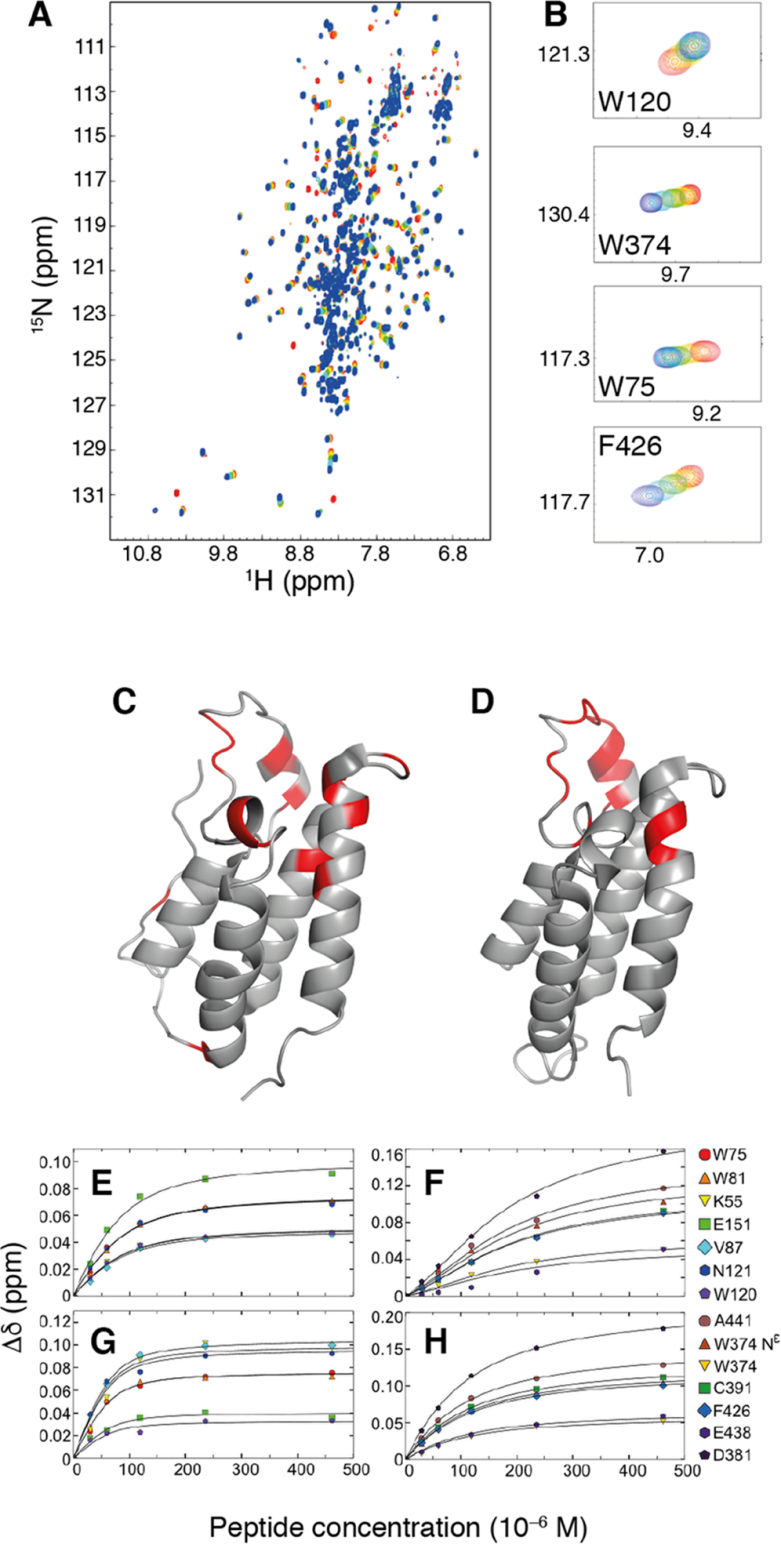
Binding of tetra-acetylated H4 peptide to the BRD4 bromodomains. (A) Superimposed ^15^N-TROSY spectra of BRD4(1,2) tracking chemical shift changes during titration with the H4Kac4 peptide. (B) Close-up views of chemical shift changes observed for four representative residues. (C, D) Backbone amide chemical shift differences Δδ > 0.1 between apo and H4Kac4-bound states of the isolated domains, colored red on the (C) BD1 structure, PDB-ID: 4CLB,^14^ and (D) on the BD2 structure, PDB-ID: 2LSP.^15^ Binding isotherms from chemical shift perturbations (Δδ) of H4Kac4 binding to BD1 (E, G) and BD2 (F, H) in tandem construct (E, F) and as isolated domains (G, H). Figure S1 shows the chemical shift differences between apo and peptide-bound states plotted versus residue number. Panels (C) and (D) were prepared using PyMOL.^16^

We investigated ligand binding to the isolated and tandem bromodomains of BRD4 by monitoring chemical shift changes during titration with a tetra-acetylated peptide comprising the N-terminal sequence of histone 4 (residues 1–16) with lysine acetylation on K5, K8, K12, and K16 (denoted H4Kac4), as shown in Figure 3. The chemical shift perturbations allowed us to identify the residues engaged in association with H4Kac4. Residues in BD1 and BD2 with chemical shift changes greater than one standard deviation above the average are primarily located in the ZA and BC loops and also in the αA, αB, and αC helices (Figure 3C,D), thereby verifying that the binding modes observed in solution generally agree with those expected from the database of available structures.^4^ The spectra further showed that the exchange between free and bound forms ranges from slow-to-intermediate to intermediate-to-fast on the chemical shift timescale, reflecting the variation in chemical shift perturbation upon peptide binding. For instance, the highly conserved residues N140 in BD1 and N433 in BD2, which are known to be key in mediating stable interactions with the ligand, appeared in the slow-exchange regime, as a consequence of their greater change in chemical shift upon binding. The majority of the BRD4 resonances affected by binding appeared in the fast-exchange regime and thus allowed straightforward tracking of the backbone amide ^1^H^N^ and ^15^N chemical shifts from the free to the bound form during the titration, as described next. We used the titration-dependent chemical shift changes for six backbone amides of BD1 (K55, W75, W81, V87, N121, and E151) and five backbone amides of BD2 (W374, D381, C391, F426, and E438), as well as the indole NH group of W374, to calculate the dissociation constant (*K*_d_) of the bromodomain–peptide interactions (Figure 3 and Table 1) using equations describing ligand binding to a single site, in the case of the isolated domains, or two sites simultaneously, in the case of the tandem domain BRD4(1,2). The NMR-derived affinities for H4Kac4 show significant differences between the two isolated domains, with *K*_d_ values of 9 μM for BD1 and 74 μM for BD2 (Figures 3 and S1 and Table 1), in good agreement with previous results.^64^ These dissociation constants can be compared with results obtained for H4 octapeptides mono-acetylated on either K5 or K16, which have mutually similar *K*_d_ values of roughly 300 and 120 μM for isolated BD1 and BD2, respectively.^65^ Similar results have been obtained for 16-mer H4 peptides mono-acetylated on K5 or K12, with *K*_d_ of 600 μM and 1 mM.^23^ Thus, the comparison confirms that multiple acetylation of the histone peptide leads to higher affinity for BRD4.^64,66^ Furthermore, higher affinity of BD1, compared to BD2, has also been observed for diacetylated transcription factor motifs.^61^ In the tandem construct BRD4(1,2), the *K*_d_ values of the individual domains binding to H4Kac4 are 23 μM for BD1 and 125 μM for BD2 (Figures 3 and S1 and Table 1).

**Table 1:** Dissociation Constants for H4Kac4-Bromodomain Complexes Measured by NMR Chemical Shift Perturbation

construct	*K*_d_(10^–6^ M) BD1	*K*_d_(10^–6^ M) BD2
isolated^a^	9 ± 1	74 ± 2
tandem^b^	23 ± 2	125 ± 10

a Determined using equations for one-site ligand binding.

b Determined using equations for two-site ligand binding.

Thus, the affinity is higher for BD1 than BD2 in both the case of the individual domains and the tandem construct. The reduction in H4Kac4 affinity of each domain in BRD4(1,2) compared to the isolated domains, amounts to a modest reduction in the free energy of binding: 2.2 kJ/mol for BD1 and 1.3 kJ/mol for BD2, which can be interpreted as unfavorable coupling free energy between the domains in BRD4(1,2). BRD4 shows higher affinity for tetra-acetylated H4 motifs than for peptides with lower levels of acetylation, which has been ascribed to avidity effects.^64^ This concept is supported by bivalent binding of a single peptide or synthetic ligand to two BDs in several crystal structures.^19,21,22,64,67,68^ However, the unfavorable coupling free energy measured for both BD1 and BD2 in the tandem construct argues against this interpretation in the case of H4Kac4 binding to BRD4(1,2). Nonetheless, bivalent binding might play a role in the case of the isolated domains because the reduced peptide affinity observed for the individual domains of BRD4(1,2) might reflect a reduced tendency to form bivalent complexes in the tandem construct, compared to the isolated domains. Furthermore, several residues exhibit differences between the isolated and tandem constructs in their chemical shift perturbations upon peptide binding, which might be explained by differential dimer formation, or possibly by interactions with the linker that might be altered by peptide binding. We return to this issue of dimerization below.

### Bromodomain Dynamics: Overview of ^15^N Relaxation Measurements

**Figure 4:**
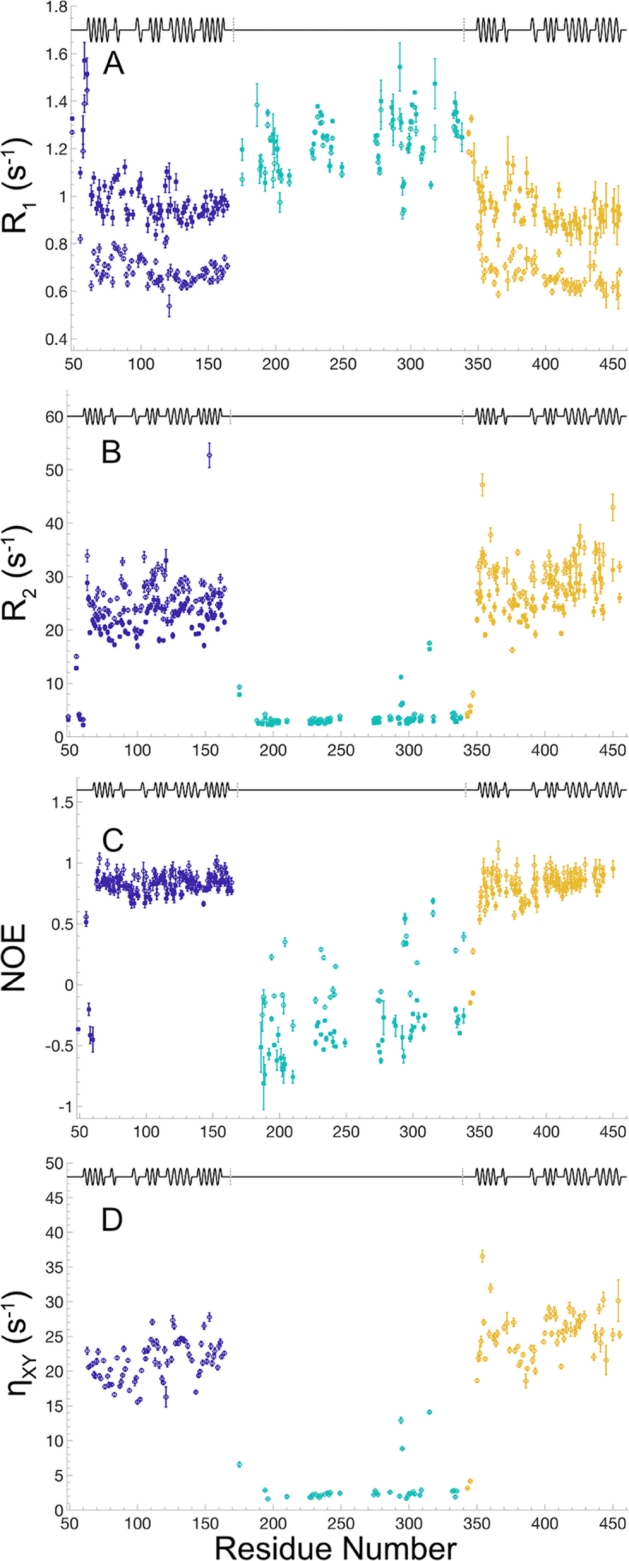
^15^N relaxation data for the tandem construct BRD4(1,2). (A) *R*_1_ relaxation rate constants, (B) *R*_2_ relaxation rate constants, (C) {^1^H}–^15^N NOEs, (D) η*_xy_* relaxation rate constants. The secondary structure is indicated at the top of each panel, with α-helixes represented by waves and loops and the linker region as straight lines. The individual domains and the linker are indicated by color: blue, BD1; teal (green), linker; and yellow, BD2. Data acquired at 14.1 and 18.8 T are indicated by filled squares and open circles, respectively.

To explore in more detail the potential domain interactions and the dynamics of the domains, we characterized the molecular dynamics of BRD4 bromodomains using ^15^N nuclear spin relaxation measurements. We performed ^15^N *R*_1_, *R*_2_, and steady-state heteronuclear {^1^H}–^15^N NOE experiments at static magnetic field strengths of 14.1 and 18.8 T to quantify picoseconds to nanosecond (ps–ns) dynamics. In addition, we acquired TROSY-based cross-correlated relaxation (η*_xy_*) experiments at 18.8 T to aid in estimating the overall rotational correlation times (τ_c_) and exchange contributions (*R*_ex_) to the transverse relaxation rates. η*_xy_*, caused by interference between ^1^H–^15^N dipole–dipole and ^15^N chemical shift anisotropy (CSA) interactions, is not affected by chemical exchange and hence provides an improved estimate of τ_c_, while comparison of *R*_2_ and η*_xy_* provides an assessment of exchange, which is essential in the present case where fast exchange between free and peptide-bound states or monomeric and dimeric species might otherwise complicate the analysis of τ_c_, as well as the internal dynamics of the individual domains. Figure 4 provides an overview of the results for apo BRD4(1,2), which clearly identifies the two domains and demonstrates that the linker region between them is highly flexible as indicated by its higher *R*_1_, lower *R*_2_, lower η*_xy_*, and lower NOE values compared to the BDs. However, we note that the linker region shows significant nonmonotonous variation in *R*_1_ and NOE among residues, indicating that it does not behave as a simple random coil-like chain, but most likely has propensity to form more ordered structure in certain regions. Figure S2 shows the corresponding results for peptide-bound BRD4(1,2), as well as for apo and peptide-bound BD1 and BD2.

### Bromodomain Dynamics: Rotational Diffusion of the BD1 and BD2 Domains

We determined the rotational diffusion tensors of the ligand-free (apo) states of BD1 and BD2 domains in both the isolated and tandem constructs. The trimmed and weighted averages of the measured relaxation rates (Table S1) give a first indication of the relative difference in overall tumbling time (τ_c_) of the different constructs. The *R*_1_ relaxation rate is proportional to 1/τ_c_, and the η*_xy_* relaxation rate is proportional to τ_c_. The *R*_2_ relaxation rate can also be used to evaluate the global diffusion time, but it includes contributions from conformational exchange on the microsecond to millisecond timescales, *R*_ex_, which complicates the analysis. For both the apo and peptide-bound forms of isolated BD2, the average values of *R*_1_ and η*_xy_* are significantly lower and higher, respectively, than the corresponding values for the isolated BD1, indicating slower global tumbling of BD2 compared to BD1. This difference persists in apo and peptide-bound BRD4(1,2), although it is slightly attenuated (Table S1 and Figure S2). The observed difference in τ_c_ is unexpected given that the isolated BD1 and BD2 domains have the same molecular weight (17.5 kDa) and similar tertiary structures consisting of α-helical bundles (Figure 1). Indeed, hydrodynamics calculations performed using HydroNMR^55,56^ predict diffusion tensors with effective τ_c_ values of 7.9 ns for BD1 and 8.3 ns for BD2 (Table 2). We estimated the diffusion tensors of the different constructs using a modified version of the rotdif program,^50^ which takes as input the measured *R*_1_, NOE, and η*_xy_* relaxation rates, thereby avoiding exchange contributions to the transverse relaxation rate. It should be noted that diffusion analysis of the bromodomains is challenging because most residues are located in α-helices with their ^15^N–^1^H bond vectors pointing along the largest principal axis of the anisotropic diffusion tensor (Figure S3), and the scarcity of bond vectors oriented perpendicular to the unique diffusion axis leads to uncertainty in the estimated values. First, we analyzed the relaxation rate constants for the isolated apo states. The results for isolated apo BD1 indicate an anisotropic diffusion tensor with *D*_∥_/*D*_⊥_ = 1.56 ± 0.02 and τ_c_ = 7.6 ± 0.9 ns, in good agreement with the value expected from the HydroNMR calculations (Table 2). By contrast, the results for isolated apo BD2 yield *D*_∥_/*D*_⊥_ = 1.39 ± 0.01 and τ_c_ = 10.1 ± 0.9 ns. Notably, τ_c_ is considerably greater than the expected value, suggesting partial dimerization of this domain. Furthermore, the lower value of *D*_∥_/*D*_⊥_ also suggests partial formation of a side-by-side dimer (which is expected to have a more spherical shape than the monomer) in line with previous hypotheses based on crystal structures^62,63^ and the concentration-dependent chemical shift changes described above. Using the dimeric structure reported for BD1 of BRD2, PDB ID 2DVQ,^63^ as a model for the tentative BD2 dimer, HydroNMR calculations predict a slightly greater value, τ_c_ = 13.8 ns, than the experimentally determined one. Using the relative populations of BD2 in monomeric (*p*_m_ = 0.66 at *P*_t_ = 135 μM) and dimeric (*p*_d_ = 0.34) states, determined from the chemical shift data, together with the monomer and dimer τ_c_ values from HydroNMR, the population-weighted average τ_c_ = 10.2 ns, in very good agreement with the experimentally determined value reported above (10.1 ns). These results provide additional evidence that isolated BD2 of BRD4 is exchanging between monomeric and dimeric states.

**Table 2:** Diffusion Tensor Parameters of BRD4 Bromodomains^a^

rotdif fitting of experimental data				
state (construct)	*D*_iso_(10^7^ s^–1^)	*D*_∥_/*D*_⊥_	τ_c_ (ns)	χ_red_^2^
apo BD1 (isolated)	2.2 ± 0.3	1.56 ± 0.02	7.6 ± 0.9	1.5
apo BD2 (isolated)	1.7 ± 0.1	1.39 ± 0.01	10.1 ± 0.9	2.5
apo BD1 (tandem)	1.3 ± 0.3	1.56 ± 0.01	13 ± 3	3.0
apo BD2 (tandem)	1.2 ± 0.3	1.59 ± 0.02	14 ± 3	4.8
H4Kac4-bound BD2 (isolated)	1.4 ± 0.1	1.59 ± 0.02	11.9 ± 0.9	2.8
H4Kac4-bound BD1 (tandem)	1.0 ± 0.3	1.69 ± 0.01	16 ± 4	3.9
H4Kac4-bound BD2 (tandem)	0.1 ± 0.4	1.92 ± 0.01	16 ± 7	5.4

HydroNMR calculations				
state (construct)	*D*_iso_(10^7^ s^–1^)	*D*_∥_/*D*_⊥_	τ_c_ (ns)	χ_red_^2^
apo BD1 (monomer)^a^	2.1	1.4	7.9	n.a.^f^
apo BD2 (monomer)^b^	2.0	1.8	8.3	n.a.^f^
H4K12ac-bound BRD2-BD1 (dimer)^c^	12	0.83	13	n.a.^f^
H4K5acK8ac-bound BD1 (monomer)^d^	2.0	1.4	8.4	n.a.^f^
H4K8acK12ac-bound BD1 (dimer)^e^	0.73	1.12	33	n.a.^f^

a PDB-ID: 4CLB.^14^

b PDB-ID: 2LSP.^15^

c PDB-ID: 2DVQ.^63^

d PDB-ID: 3UVW.^64^

e PDB-ID: 3UW9.^63^

f Not applicable.

**Figure 5:**
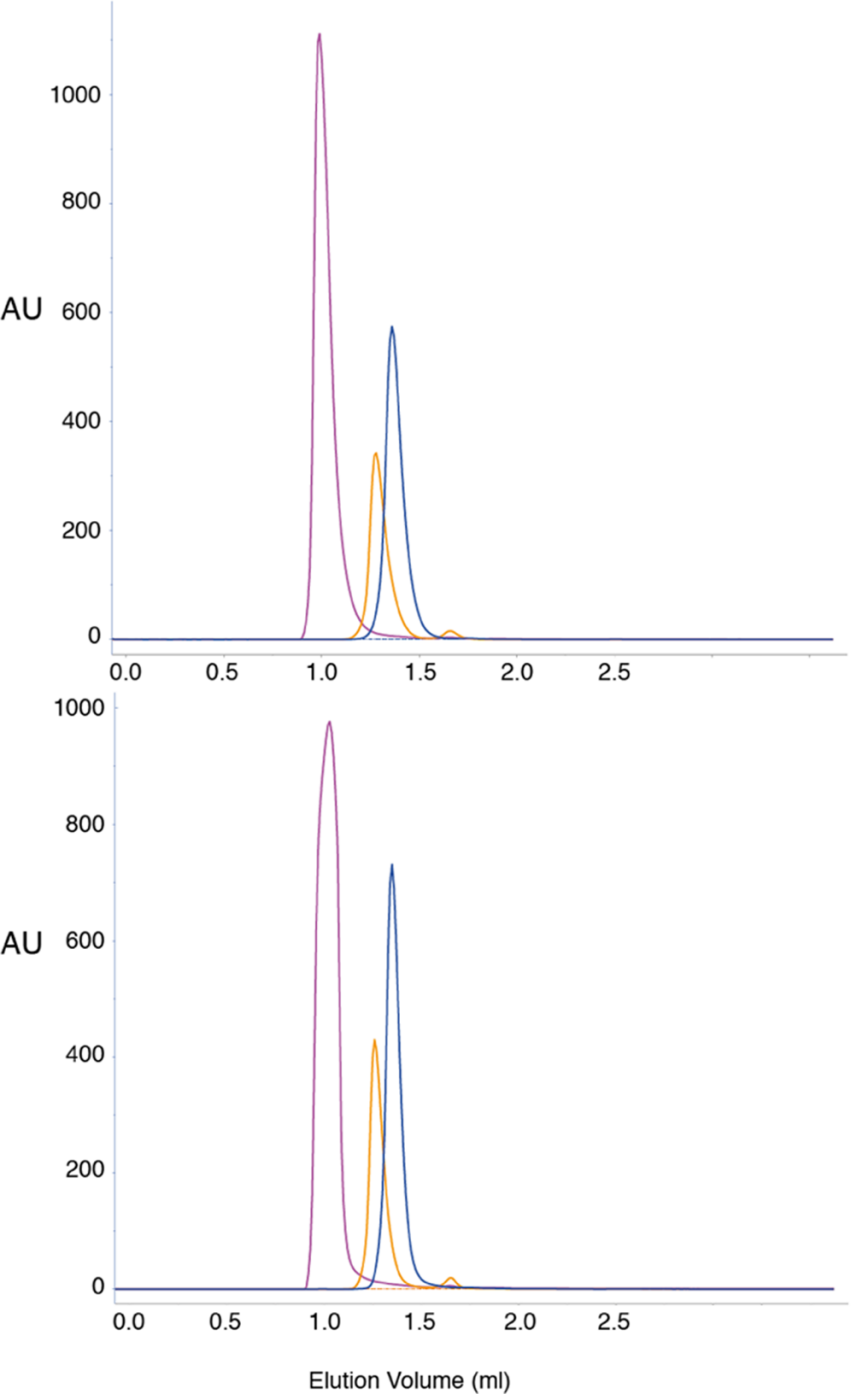
Size exclusion chromatography of isolated BD1 (blue) and BD2 (yellow) together with tandem BRD4(1,2) (magenta). The top and bottom panels show the elution chromatogram for sample concentrations of 125 and 350 μM, respectively. The retention times are identical in the two panels for BD1, corresponding to an *M*_w_ of 18 kDa, whereas minute differences are observed between the two panels for BD2, corresponding to differences in measured *M*_w_ of 25 versus 28 kDa. The calculated *M*_w_ is 17.5 kDa for both BD1 and BD2.

We validated the results for the apo forms of isolated BD1 and BD2 by size exclusion chromatography (SEC), which showed that isolated apo BD1 is monomeric, whereas isolated apo BD2 elutes as a larger protein than expected and this effect is very slightly pronounced at higher concentration, consistent with partial dimerization (Figure 5). The result for BD2 contrasts with previous interpretations of SEC data for the wild-type and mutant forms, designed to disrupt the dimer interface, which suggested that BD2 is monomeric despite eluting as a larger species;^65^ this study also did not detect any signs of heterodimer formation between BD1 and BD2. Previous ^15^N NMR relaxation results have also suggested that BD2 has a greater hydrodynamic radius than does BD1 (in agreement with our results), but analytical ultracentrifugation experiments performed in the same study indicated that the domain is monomeric.^23^ However, we note that analytical ultracentrifugation was conducted with significantly lower protein concentrations (by factors of 2–10) than those used in the NMR study. In addition, differences in sequence length (construct size) among the studied systems could play a role. For example, Liu et al. used a shorter construct of BD2, comprising residues 352–457 (compared to our version comprising 341–460), which lacks residues next to the proposed dimer interface.^23^

Next, we characterized the rotational diffusion properties of the domains in the context of the tandem construct, BRD4(1,2). The best-fit diffusion tensors of the individual domains in BRD4(1,2) are *D*_∥_/*D*_⊥_ = 1.56 ± 0.01 and τ_c_ = 13 ± 3 ns for BD1, and *D*_∥_/*D*_⊥_ = 1.59 ± 0.02 and τ_c_ = 14 ± 3 ns for BD2. The anisotropy of each domain in BRD4(1,2) is indistinguishable from that of the isolated BD1 domain, indicating that partial dimer formation of BD2 is significantly reduced in the tandem construct, compared to the isolated domain, or altogether abolished. The higher value of τ_c_ for BD1 in BRD4(1,2) compared to the isolated domain is explained by the motional restriction imparted by the connection of the two domains via the linker.^69,70^ The slightly greater τ_c_ value for the BD2 domain, compared to BD1, in BRD4(1,2) is in line with the difference in τ_c_ values predicted by HydroNMR for the isolated domains.

We studied the effect of peptide binding on the rotational diffusion of the bromodomains. In the H4Kac4-bound state, the isolated BD2 domain has *D*_∥_/*D*_⊥_ = 1.59 ± 0.02 and τ_c_ = 11.9 ± 0.9 ns, reflecting a modest increase in τ_c_ compared to the partially dimeric apo state. We did not record η*_xy_* for isolated peptide-bound BD1, precluding the rotational diffusion analysis described above, but model-free analysis (based on *R*_1_, *R*_2_, and NOE; see below) yields *D*_∥_/*D*_⊥_ = 1.8 and τ_c_ = 12.3 ns, similar to the results for BD2 (Table S2). These results might suggest that the tetra-acetylated peptide binds to the isolated domains in a bivalent mode, thereby inducing dimerization. As noted above, a number of crystal structures of bromodomains have revealed bivalent binding of peptides^64,67,68^ and synthetic inhibitors.^19,21,22^ These structures show a great deal of variation in the relative orientation of the two domains, including side-by-side and fully extended head-to-head orientations. In all cases, the τ_c_ values predicted by HydroNMR (Table 2) for these dimers are considerably greater than the experimentally determined one, indicating that the peptide-bound form of isolated BD1 dimerizes transiently, similar to isolated BD2 (which apparently does so in both the apo and bound forms). The partial dimerization observed in the presence of peptide might be the result of bivalent peptide binding to two bromodomains.

In the tandem construct, the peptide-bound BD1 and BD2 domains are characterized by *D*_∥_/*D*_⊥_ = 1.69 ± 0.01 and τ_c_ = 16 ± 4 ns and *D*_∥_/*D*⊥ = 1.92 ± 0.01 and τ_c_ = 17 ± 7 ns, respectively. The relatively large uncertainties in these results unfortunately preclude any firm assessment of potential dimer formation in this case.

### Internal Dynamics of BD1 and BD2 and Effects of Peptide Binding

We analyzed the internal dynamics on the ps–ns timescale using the model-free (MF) formalism^45−47^ and the dynamic effects of binding H4Kac4 to each domain. The MF analysis included the order parameters (*S*^2^, *S*_f_^2^) and effective correlation times (τ_e_, τ_s_) of the sub-ns internal motion, together with the overall rotational diffusion (τ_c_ and *D*_∥_/*D*_⊥_), while slower motions were treated simply as exchange contributions (*R*_ex_) to *R*_2_. MF analysis of the bromodomains is hampered to some extent by the limited range of ^15^N–^1^H bond vector orientations sampling the diffusion tensor, as described above. Furthermore, exchange between monomeric and dimeric states results in population-weighted averages of relaxation rates associated with the two different diffusion tensors,^71^ which presents a potential caveat for the MF analysis of BD2 since the detailed structure of the dimer is unknown. However, three reasonable assumptions make the analysis tractable: first, the N–H bond vector orientations in the molecular frame do not change upon dimer formation; second, the order parameter is identical in the monomer and dimer; and third, the diffusion tensor of the dimer is nearly isotropic. With these assumptions, MF analysis can be performed on BD2, while recognizing that the determined diffusion tensor principal values represent an effective apparent tensor. The MF analysis is further dependent on the quality of the structural models because errors in the N–H bond vector orientations in the principal axis frame of the diffusion tensor affect the fitted MF parameters and often translate into artificial *R*_ex_ values in the range of 1–3 s^–1^. To assess the impact of these effects on the fitted order parameters, we also performed MF fits using an effective correlation time (local τ_m_) for each residue, without reference to the overall structure of the protein. The resulting two sets of MF parameters determined by these alternative approaches generally agree well with a mean deviation in *S*^2^ of less than 0.03 ± 0.02 for all states and constructs.

**Figure 6:**
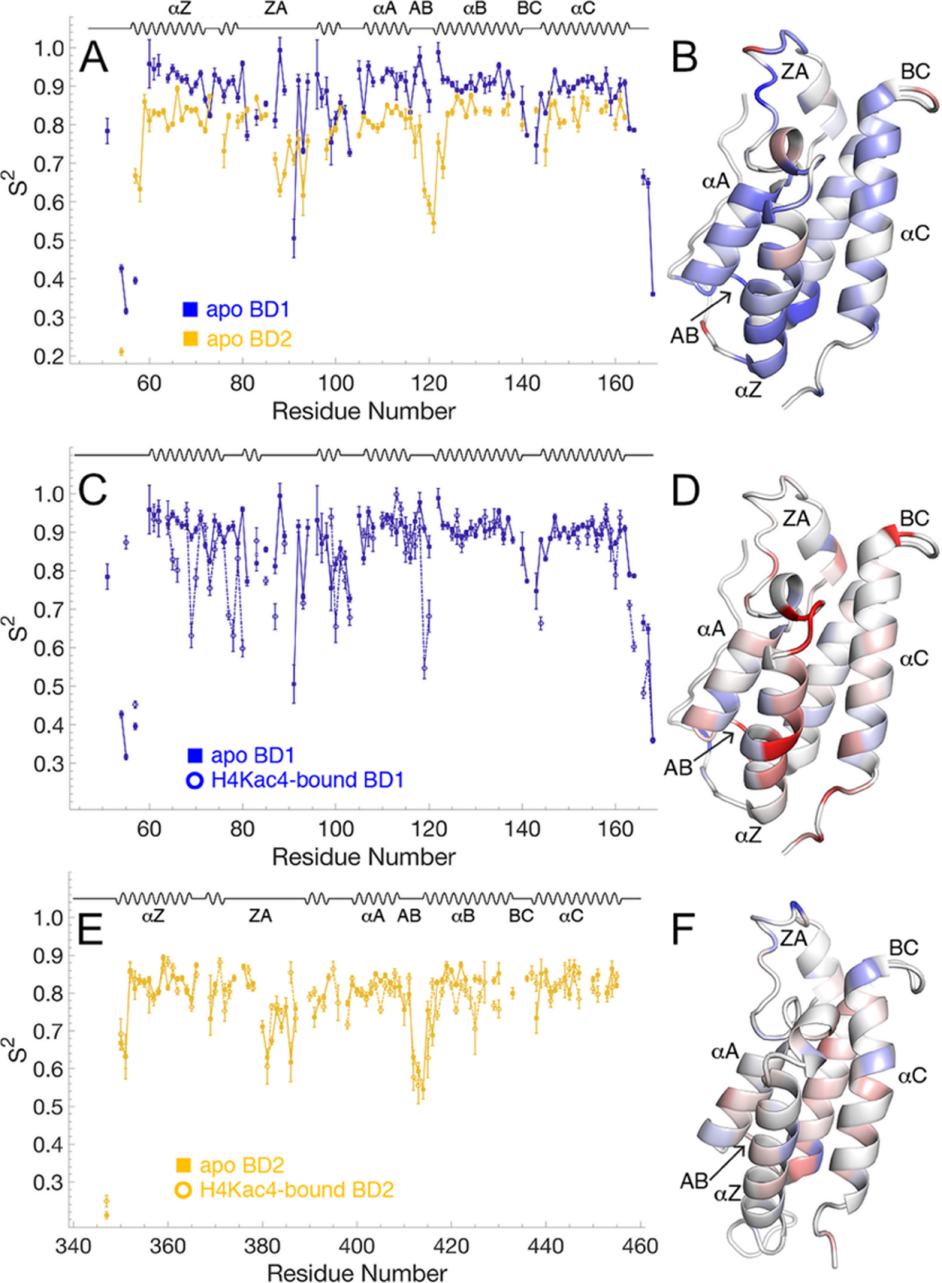
NMR order parameters (*S*^2^) of isolated BRD4 bromodomains in the apo and H4 tetra-acetylated peptide (H4Kac4)-bound states. (A) *S*^2^ versus residue number for the apo states of BD1 (blue) and BD2 (yellow). The sequences are aligned via the BC loop segment (residues 140–144 in BD1, 433–437 in BD2). (B) Difference in *S*^2^ between apo BD1 and BD2 (data from panel (A)), color-coded onto the BD1 structure, PDB: 4CLB.^14^ (C) *S*^2^ versus residue number for BD1, apo (filled squares, full line), H4Kac4-bound (open circles, dashed line). (D) Difference in *S*^2^ between H4Kac4-bound and apo BD1, color-coded onto the BD1 structure. (E) *S*^2^ versus residue number for BD2, apo (filled squares, full line), H4Kac4-bound (open circles, dashed line). (F) Difference in *S*^2^ between H4Kac4-bound and apo BD2, color-coded onto the BD2 structure, PDB: 2LSP.^15^ The black line at the top of panels (A), (C), and (E) indicates the location of loops (lines) and α-helices (waves); in panel (A), the line refers to BD1. The color coding in panels (B), (D), and (F) depicts differences in *S*^2^: (B) Δ*S*^2^ = *S*^2^(apo BD1) – *S*^2^(apo BD2); (D, F) Δ*S*^2^ = *S*^2^(H4Kac4-bound) – *S*^2^(apo), in the range [−0.3; 0.3] from red (negative), via white to blue (positive). Panels (B), (D), and (F) were prepared using PyMOL.^16^

**Figure 7:**
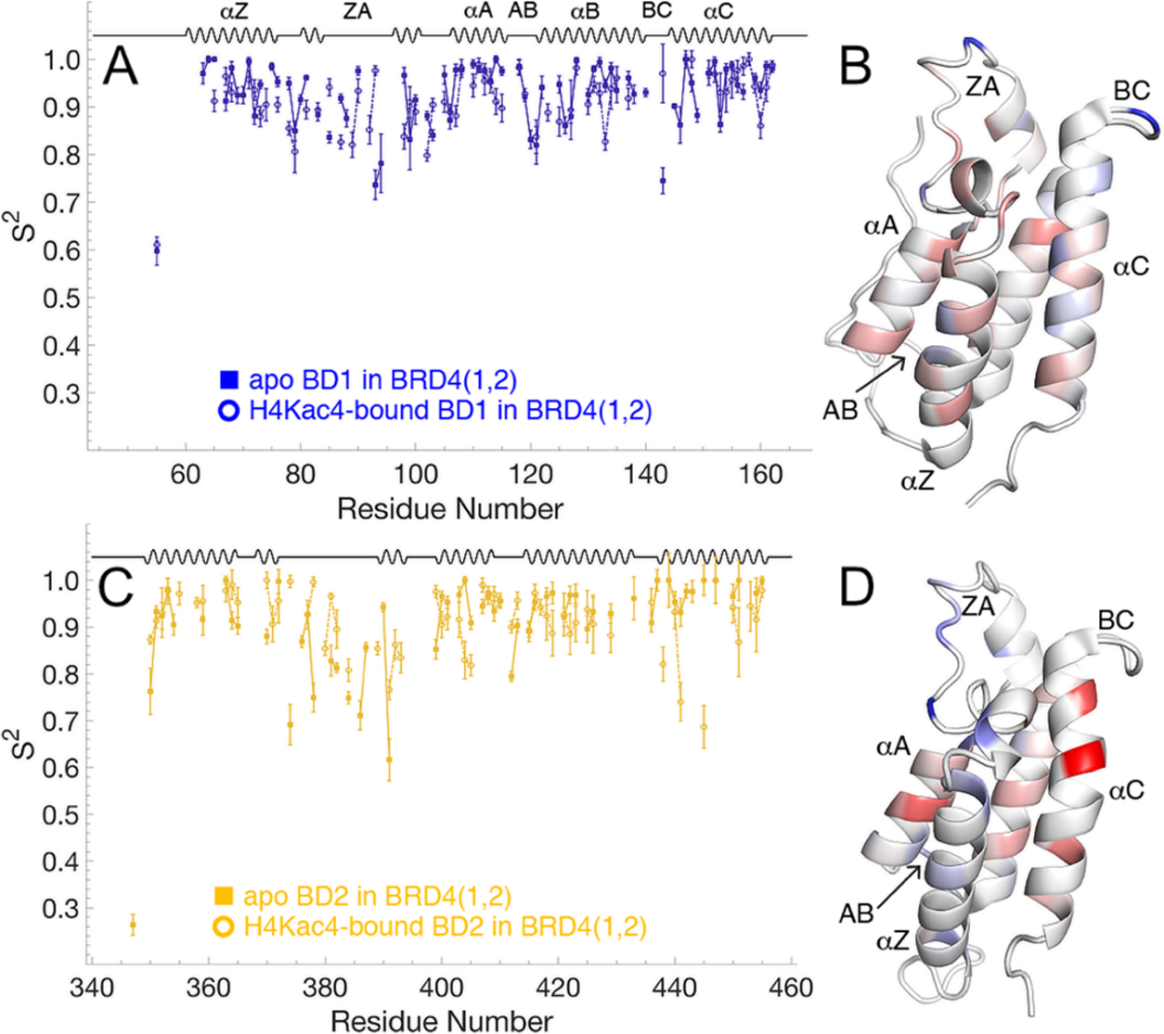
NMR order parameters (*S*^2^) of tandem BRD4 bromodomains in the apo and H4Kac4-bound states. (A) *S*^2^ versus residue number for BD1, apo (filled squares), H4Kac4-bound (open circles). (B) Difference in *S*^2^ between H4Kac4-bound and apo BD1, color-coded onto the BD1 structure, PDB: 4CLB.^14^ (C) *S*^2^ versus residue number for BD2, apo (filled squares) and H4Kac4-bound (open circles). (D) Difference in *S*^2^ between H4Kac4-bound and apo BD2, color-coded onto the BD2 structure, PDB: 2LSP.^15^ The black line at the top of panels (A) and (C) indicates the location of loops (lines) and α-helices (wave). The color coding in panels (C) and (D) depicts differences in *S*^2^, Δ*S*^2^ = *S*^2^(H4Kac4-bound) – *S*^2^(apo), in the range [−0.3; 0.3] from red (negative), via white to blue (positive). Panels (B) and (D) were prepared using PyMOL.^16^

The MF optimization generally resulted in back-calculated relaxation rates that are in good agreement with the experimental data (full set of fitted MF parameters and back-calculated relaxation rates are available via the BMRB; accession numbers 51413–51418). Overall, the resulting estimates of the global diffusion tensors (Table S2) appear to be fully consistent with the results from rotdif (Table 2) and SEC (Figure 5), indicating a successful separation of global and local motions in the MF analysis. The final order parameters resulting from the MF analysis are shown in Figures 6 and 7.

We investigated how the internal dynamics differ between BD1 and BD2 in their isolated and tandem forms, and how each domain responds to ligand binding. We initially focus on the results for the isolated domains because the underlying data are generally of higher quality compared to those for the tandem construct. The average order parameter of residues in α-helices differs between the two domains in the apo state (Figure 6A,B), with values of 0.90 for BD1 and 0.82 for BD2 (the standard error of the mean is less than 0.01 in each case), which can be compared with the average value of 0.88 ± 0.07 (1 standard deviation) for residues in α-helices, obtained from a larger database of *S*^2^ values in globular proteins.^72^ The observed difference in *S*^2^ indicates that BD1 is more rigid than BD2, a result that is in general agreement with recent molecular dynamics (MD) simulations^8,73^ and amide-exchange mass-spectrometry.^74^ Furthermore, the overall stability toward unfolding in urea is also markedly different, with BD1 being more stable than BD2 toward loss of tertiary structure.^75^

The two domains show different profiles of *S*^2^ values along the protein sequence, where in particular the ZA and AB loops have higher mobility in BD2, whereas the BC loop has similar mobility in the two domains (Figure 6A,B). Two recent MD simulations both indicate a higher ZA mobility in BD2, as well as similar fluctuations of the BC loop in the two domains,^8,73^ but only the study by Cheng et al.^8^ shows an effect on the AB loop similar to our results.

Figure 6C–F shows comparisons of the order parameters for the apo and peptide-bound states of the two isolated domains. Upon peptide binding BD1 gains flexibility relative to the apo form (Figure 6C,D), specifically in the ZA and AB loops, and apparently also in the BC loop, although there are few data points in this loop for the peptide-bound state. In BD2, peptide binding leads to lower-order parameters in the ZA and BC loops, but not in the AB loop (Figure 6E,F). As mentioned above, MD simulations have suggested that binding of various synthetic ligands can lead to increased conformational fluctuations of the bromodomains, where the relative changes in BD1 and BD2 depend sensitively on the ligand structure. Our present results now detail the response of the BDs to binding a natural H4Kac4 peptide. Increased flexibility of the loop segments in the peptide-bound state suggests that the structure becomes slightly more expanded with increased hydrodynamic radius, which is in agreement with the results on rotational diffusion described above. The difference in flexibility of the AB loop is highly unexpected since it is located at the opposite end of the four-helix bundle from the binding site, but its internal dynamics might reflect dimerization. The *S*^2^ values are low for the AB loop in apo BD2, which is exchanging between monomeric and dimeric states. Peptide binding to BD2 does not change the *S*^2^ values of the AB loop and it does not appear to change the population of dimers. Apo BD1 has high *S*^2^ values in the AB loop and it is monomeric. Peptide binding to BD1 leads to partial dimerization and reduction in the *S*^2^ values of the AB loop. Thus, increased flexibility of the AB loop is likely the result of dimer formation.

Taken together, the order parameters show that the dynamic response to binding a natural acetylated peptide varies significantly between BD1 and BD2, demonstrating that the detailed amino acid sequence has dramatic consequences on the internal dynamics as well as the propensity to form dimers, despite the high degree of structural homology between the domains (cf. Figure 1).

Compared to their isolated forms, both domains of BRD4(1,2) appear to be more rigid regardless of whether they are in the apo or peptide-bound states (Figure 7). In the tandem construct the BD1 domain shows relatively small changes in order parameters between the apo and peptide-bound states, indicating limited structural-dynamical changes upon binding. This result is in agreement with the small changes in the diffusion tensor reported above and indicates that bivalent peptide binding occurs to a much lower extent in the tandem construct than in the isolated domain. The differences in order parameters between the apo and peptide-bound states of BD2 in BRD4(1,2) seem to indicate that peptide binding leads to slightly decreased flexibility of the ZA loop, but increased flexibility of the BC loop, whereas the AB loop is less affected.

### Interpreting Slower Timescale Exchange Dynamics

**Figure 8:**
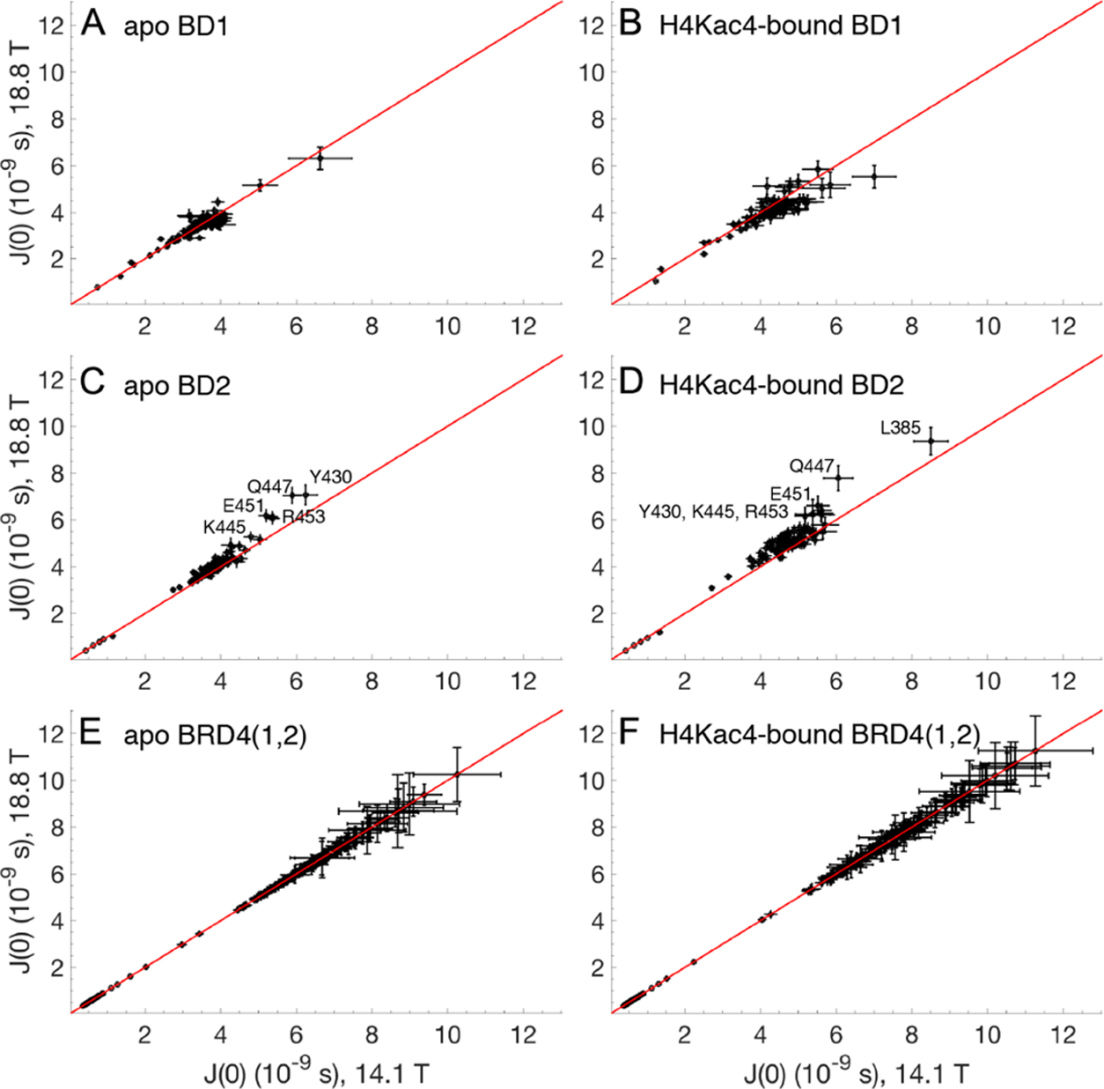
Spectral density values *J*(0) determined by spectral density mapping of ^15^N relaxation data measured at static magnetic field strengths of 14.1 and 18.8 T: (A) apo BD1, (B) H4Kac4-bound BD1, (C) apo BD2, (D) H4Kac4-bound BD2, (E) apo BRD4(1,2), (F) H4Kac4-bound BRD4(1,2). Black symbols show pairs of *J*(0) determined at *B*_0_ = 14.1 and 18.8 T with error bars indicating one standard deviation. The red line, with a slope of 1 and intercept of 0, is drawn to guide the eye.

The MF analysis results in conformational exchange contributions, *R*_ex_, to the transverse relaxation rates for a relatively large number of residues, especially in BD2. To validate these results, we performed spectral density mapping,^58^ based on the *R*_1_, *R*_2_, and NOE relaxation parameters. In the absence of exchange contributions to *R*_2_, the spectral density component *J*(0) should not depend on the static magnetic field strength (*B*_0_). In the presence of exchange on the intermediate to fast timescale, spectral density mapping instead results in increased *J*(0) values with increasing *B*_0_. Thus, by plotting the *J*(0) values extracted from the relaxation datasets obtained at 14.1 and 18.8 T against one another, we identified those residues that deviate from the straight line with unit slope and zero intercept as likely to experience exchange (Figure 8). This analysis clearly indicates that isolated BD2 shows exchange in both the apo and H4Kac4-bound states, in agreement with the results presented above for concentration-dependent chemical shifts and population-weighted τ_c_ values, which indicate exchange between monomeric and dimeric states. In fact, residues forming dimer contacts, e.g., Y430, K445, Q447, and E451, are among those that exhibit the largest *J*(0) values and deviate the most from the straight line in Figure 8C,D. In contrast, the exchange is less prominent in isolated BD1 and essentially absent in BRD4(1,2) for both domains. To further validate the MF-derived *R*_ex_ terms for isolated BD2, we compared these with exchange contributions estimated by comparing Γ_auto_ and Γ_cross_, determined from linear combinations of relaxation rate constants involving either *R*_2_ or η*_xy_*, respectively, using the approach presented by Palmer and co-workers;^53,59^ see the Materials and Methods section. This analysis confirms the larger *R*_ex_ contributions estimated by the MF approach for isolated BD2 in the apo and peptide-bound states (data not shown), as also indicated by Figure 8C,D. There is no correlation between Δδ and *R*_ex_ determined for the peptide-bound state of BD2, indicating that the exchange is not due to exchange kinetics between free and bound states, but rather reflects monomer–dimer exchange and intrinsic conformational dynamics on the micro- to millisecond timescale.

## Concluding Remarks

We have investigated the dynamics of the individual bromodomains of BRD4 and their interactions with a tetra-acetylated peptide from histone 4, both in the context of tandem BRD4(1,2) and as isolated domains. We have identified notable differences between BD1 and BD2 in their propensities to form dimers, in their dynamics, and in the response of these characteristics to peptide binding. These results establish a basis for understanding the role of intrinsic bromodomain dynamics in governing interactions with acetylated histones and transcription factors, which in many cases seem to involve cooperative bromodomain binding.^4^ Furthermore, the present paper describes differential intramolecular dynamics of the two bromodomains that should provide valuable insights relevant to drug design initiatives to achieve inhibitor selectivity.

## Supporting Information Available

Relaxation data, difference plots comparing chemical shifts and order parameters of different states, histograms of N–H bond vector distributions, and trimmed means of relaxation datasets (PDF)

## Accession Codes

Uniprot: O60885. Biological Magnetic Resonance Data Bank (BMRB): 51413, 51414, 51415, 51416, 51417, 51418.

## Abbreviations

BET: bromodomain and extra-terminal protein
BRD4: bromodomain-containing protein 4
BD1: bromodomain 1
BD2: bromodomain 2
CSA: chemical shift anisotropy
DSS: 4,4-dimethyl-4-silapentane-1-sulfonic acid
H4Kac4: tetra-acetylated peptide comprising the N-terminal sequence of histone 4 (residues 1–16) with lysine acetylation on K5, K8, K12, and K16
HEPES: 4-(2-hydroxyethyl)piperazine-1-ethanesulfonic acid
HMQC: heteronuclear multiple-quantum coherence
Kac: acetylated lysine residue
MF: model-free
MD: molecular dynamics
NMR: nuclear magnetic resonance
NOE: nuclear Overhauser enhancement
SEC: size exclusion chromatography
TCEP: tris(2-carboxyethyl)phosphine
TEV: tobacco etch virus
TROSY: transverse relaxation optimized spectroscopy

## References

[ref1] Taverna S. D.; Li H.; Ruthenburg A. J.; Allis C. D.; Patel D. J. How chromatin-binding modules interpret histone modifications: lessons from professional pocket pickers. Nat. Struct. Mol. Biol. 2007, 14, 1025–1040. 10.1038/nsmb1338. 17984965 PMC4691843

[ref2] Dhalluin C.; Carlson J. E.; Zeng L.; He C.; Aggarwal A. K.; Zhou M.-M.; Zhou M.-M. Structure and ligand of a histone acetyltransferase bromodomain. Nature 1999, 399, 491–496. 10.1038/20974. 10365964

[ref3] Shi J.; Vakoc C. R. The Mechanisms behind the Therapeutic Activity of BET Bromodomain Inhibition. Mol. Cell 2014, 54, 728–736. 10.1016/j.molcel.2014.05.016. 24905006 PMC4236231

[ref4] Zaware N.; Zhou M.-M. Bromodomain biology and drug discovery. Nat. Struct. Mol. Biol. 2019, 26, 870–879. 10.1038/s41594-019-0309-8. 31582847 PMC6984398

[ref5] Nicodeme E.; Jeffrey K. L.; Schaefer U.; Beinke S.; Dewell S.; Chung C.; Chandwani R.; Marazzi I.; Wilson P.; Coste H.; White J.; Kirilovsky J.; Rice C. M.; Lora J. M.; Prinjha R. K.; Lee K.; Tarakhovsky A. Suppression of inflammation by a synthetic histone mimic. Nature 2010, 468, 1119–1123. 10.1038/nature09589. 21068722 PMC5415086

[ref6] Filippakopoulos P.; Qi J.; Picaud S.; Shen Y.; Smith W. B.; Fedorov O.; Morse E. M.; Keates T.; Hickman T. T.; Felletar I.; Philpott M.; Munro S.; McKeown M. R.; Wang Y.; Christie A. L.; West N.; Cameron M. J.; Schwartz B.; Heightman T. D.; Thangue N. L.; French C. A.; Wiest O.; Kung A. L.; Knapp S.; Bradner J. E. Selective inhibition of BET bromodomains. Nature 2010, 468, 1067–1073. 10.1038/nature09504. 20871596 PMC3010259

[ref7] Gilan O.; Rioja I.; Knezevic K.; Bell M. J.; Yeung M. M.; Harker N. R.; Lam E. Y. N.; Chung C.; Bamborough P.; Petretich M.; Urh M.; Atkinson S. J.; Bassil A. K.; Roberts E. J.; Vassiliadis D.; Burr M. L.; Preston A. G. S.; Wellaway C.; Werner T.; Gray J. R.; Michon A. M.; Gobbetti T.; Kumar V.; Soden P. E.; Haynes A.; Vappiani J.; Tough D. F.; Taylor S.; Dawson S. J.; Bantscheff M.; Lindon M.; Drewes G.; Demont E. H.; Daniels D. L.; Grandi P.; Prinjha R. K.; Dawson M. A. Selective targeting of BD1 and BD2 of the BET proteins in cancer and immunoinflammation. Science 2020, 368, 387–394. 10.1126/science.aaz8455. 32193360 PMC7610820

[ref8] Cheng C.; Diao H.; Zhang F.; Wang Y.; Wang K.; Wu R. Deciphering the mechanisms of selective inhibition for the tandem BD1/BD2 in the BET-bromodomain family. Phys. Chem. Chem. Phys. 2017, 19, 23934–23941. 10.1039/C7CP04608A. 28849824

[ref9] Raich L.; Meier K.; Günther J.; Christ C. D.; Noé F.; Olsson S. Discovery of a hidden transient state in all bromodomain families. Proc. Natl. Acad. Sci. U.S.A. 2021, 118, e2017427118 10.1073/pnas.2017427118. 33468647 PMC7848705

[ref10] Steiner S.; Magno A.; Huang D.; Caflisch A. Does bromodomain flexibility influence histone recognition?. FEBS Lett. 2013, 587, 2158–2163. 10.1016/j.febslet.2013.05.032. 23711371

[ref11] Zhang X.; Chen K.; Wu Y.-D.; Wiest O. Protein dynamics and structural waters in bromodomains. PLoS One 2017, 12, e0186570 10.1371/journal.pone.0186570. 29077715 PMC5659604

[ref12] Picaud S.; Wells C.; Felletar I.; Brotherton D.; Martin S.; Savitsky P.; Diez-Dacal B.; Philpott M.; Bountra C.; Lingard H.; Fedorov O.; Müller S.; Brennan P. E.; Knapp S.; Filippakopoulos P. RVX-208, an inhibitor of BET transcriptional regulators with selectivity for the second bromodomain. Proc. Natl. Acad. Sci. U.S.A. 2013, 110, 19754–19759. 10.1073/pnas.1310658110. 24248379 PMC3856850

[ref13] Raux B.; Voitovich Y.; Derviaux C.; Lugari A.; Rebuffet E.; Milhas S.; Priet S.; Roux T.; Trinquet E.; Guillemot J. C.; Knapp S.; Brunel J. M.; Fedorov A. Y.; Collette Y.; Roche P.; Betzi S.; Combes S.; Morelli X. Exploring Selective Inhibition of the First Bromodomain of the Human Bromodomain and Extra-terminal Domain (BET) Proteins. J. Med. Chem. 2016, 59, 1634–1641. 10.1021/acs.jmedchem.5b01708. 26735842

[ref14] Atkinson S. J.; Soden P. E.; Angell D. C.; Bantscheff M.; Chung C.; Giblin K. A.; Smithers N.; Furze R. C.; Gordon L.; Drewes G.; Rioja I.; Witherington J.; Parr N. J.; Prinjha R. K. The structure based design of dual HDAC/BET inhibitors as novel epigenetic probes. MedChemComm 2014, 5, 342–351. 10.1039/C3MD00285C.

[ref15] Zhang G.; Liu R.; Zhong Y.; Plotnikov A. N.; Zhang W.; Zeng L.; Rusinova E.; Gerona-Nevarro G.; Moshkina N.; Joshua J.; Chuang P. Y.; Ohlmeyer M.; He J. C.; Zhou M.-M. Down-regulation of NF-κB Transcriptional Activity in HIV-associated Kidney Disease by BRD4 Inhibition. J. Biol. Chem. 2012, 287, 28840–28851. 10.1074/jbc.M112.359505. 22645123 PMC3436579

[ref16] The PyMOL Molecular Graphics System; Schrödinger, LLC.

[ref17] Baud M. G. J.; Lin-Shiao E.; Cardote T.; Tallant C.; Pschibul A.; Chan K.-H.; Zengerle M.; Garcia J. R.; Kwan T. T.-L.; Ferguson F. M.; Ciulli A. A bump-and-hole approach to engineer controlled selectivity of BET bromodomain chemical probes. Science 2014, 346, 638–641. 10.1126/science.1249830. 25323695 PMC4458378

[ref18] Schröder S.; Cho S.; Zeng L.; Zhang Q.; Kaehlcke K.; Mak L.; Lau J.; Bisgrove D.; Schnoelzer M.; Verdin E.; Zhou M.-M.; Ott M. Two-pronged Binding with Bromodomain-containing Protein 4 Liberates Positive Transcription Elongation Factor b from Inactive Ribonucleoprotein Complexes. J. Biol. Chem. 2012, 287, 1090–1099. 10.1074/jbc.M111.282855. 22084242 PMC3256921

[ref19] Ren C.; Zhang G.; Han F.; Fu S.; Cao Y.; Zhang F.; Zhang Q.; Meslamani J.; Xu Y.; Ji D.; Cao L.; Zhou Q.; Cheung K.; Sharma R.; Babault N.; Yi Z.; Zhang W.; Walsh M. J.; Zeng L.; Zhou M.-M. Spatially constrained tandem bromodomain inhibition bolsters sustained repression of BRD4 transcriptional activity for TNBC cell growth. Proc. Natl. Acad. Sci. U.S.A. 2018, 115, 7949–7954. 10.1073/pnas.1720000115. 30012592 PMC6077712

[ref20] Wang R.; Li Q.; Helfer C. M.; Jiao J.; You J. Bromodomain Protein Brd4 Associated with Acetylated Chromatin Is Important for Maintenance of Higher-order Chromatin Structure. J. Biol. Chem. 2012, 287, 10738–10752. 10.1074/jbc.M111.323493. 22334664 PMC3322821

[ref21] Waring M. J.; Chen H.; Rabow A. A.; Walker G.; Bobby R.; Boiko S.; Bradbury R. H.; Callis R.; Clark E.; Dale I.; Daniels D. L.; Dulak A.; Flavell L.; Holdgate G.; Jowitt T. A.; Kikhney A.; McAlister M.; Méndez J.; Ogg D.; Patel J.; Petteruti P.; Robb G. R.; Robers M. B.; Saif S.; Stratton N.; Svergun D. I.; Wang W.; Whittaker D.; Wilson D. M.; Yao Y. Potent and selective bivalent inhibitors of BET bromodomains. Nat. Chem. Biol. 2016, 12, 1097–1104. 10.1038/nchembio.2210. 27775716

[ref22] Tanaka M.; Roberts J. M.; Seo H.-S.; Souza A.; Paulk J.; Scott T. G.; DeAngelo S. L.; Dhe-Paganon S.; Bradner J. E. Design and characterization of bivalent BET inhibitors. Nat. Chem. Biol. 2016, 12, 1089–1096. 10.1038/nchembio.2209. 27775715 PMC5117811

[ref23] Liu Y.; Wang X.; Zhang J.; Huang H.; Ding B.; Wu J.; Shi Y. Structural Basis and Binding Properties of the Second Bromodomain of Brd4 with Acetylated Histone Tails. Biochemistry 2008, 47, 6403–6417. 10.1021/bi8001659. 18500820

[ref24] Findeisen M.; Brand T.; Berger S. A 1H-NMR thermometer suitable for cryoprobes. Magn. Reson. Chem. 2007, 45, 175–178. 10.1002/mrc.1941. 17154329

[ref25] Markley J. L.; Bax A.; Arata Y.; Hilbers C. W.; Kaptein R.; Sykes B. D.; Wright P. E.; Wüthrich K. Recommendations for the presentation of NMR structures of proteins and nucleic acids. J. Mol. Biol. 1998, 280, 933–952. 10.1006/jmbi.1998.1852. 9671561

[ref26] Cavanagh J.; Fairbrother W. J.; Palmer A. G.; Rance M.; Skelton N. J. Protein NMR Spectroscopy Principles and Practice, 2nd ed.; Elsevier Academic Press: London, 2007.

[ref27] Schanda P.; Brutscher B. Very Fast Two-Dimensional NMR Spectroscopy for Real-Time Investigation of Dynamic Events in Proteins on the Time Scale of Seconds. J. Am. Chem. Soc. 2005, 127, 8014–8015. 10.1021/ja051306e. 15926816

[ref28] Kern T.; Schanda P.; Brutscher B. Sensitivity-enhanced IPAP-SOFAST-HMQC for fast-pulsing 2D NMR with reduced radiofrequency load. J. Magn. Reson. 2008, 190, 333–338. 10.1016/j.jmr.2007.11.015. 18078771

[ref29] Grzesiek S.; Bax A. An efficient experiment for sequential backbone assignment of medium-sized isotopically enriched proteins. J. Magn. Reson. (1969) 1992, 99, 201–207. 10.1016/0022-2364(92)90169-8.

[ref30] Grzesiek S.; Bax A. Correlating backbone amide and side chain resonances in larger proteins by multiple relayed triple resonance NMR. J. Am. Chem. Soc. 1992, 114, 6291–6293. 10.1021/ja00042a003.

[ref31] Bax A.; Ikura M. An efficient 3D NMR technique for correlating the proton and 15N backbone amide resonances with the α-carbon of the preceding residue in uniformly 15N/13C enriched proteins. J. Biomol. NMR 1991, 1, 99–104. 10.1007/BF01874573. 1668719

[ref32] Kay L. E.; Ikura M.; Tschudin R.; Bax A. Three-dimensional triple-resonance NMR spectroscopy of isotopically enriched proteins. J. Magn. Reson. (1969) 1990, 89, 496–514. 10.1016/0022-2364(90)90333-5. 22152361

[ref33] Salzmann M.; Wider G.; Pervushin K.; Wüthrich K. Improved sensitivity and coherence selection for [15N,1H]-TROSY elements in triple resonance experiments. J. Biomol. NMR 1999, 15, 181–184. 10.1023/A:1008358030477. 10605091

[ref34] Yoshimura Y.; Kulminskaya N. V.; Mulder F. A. A. Easy and unambiguous sequential assignments of intrinsically disordered proteins by correlating the backbone 15N or 13C′ chemical shifts of multiple contiguous residues in highly resolved 3D spectra. J. Biomol. NMR 2015, 61, 109–121. 10.1007/s10858-014-9890-7. 25577242

[ref35] Hyberts S. G.; Milbradt A. G.; Wagner A. B.; Arthanari H.; Wagner G. Application of iterative soft thresholding for fast reconstruction of NMR data non-uniformly sampled with multidimensional Poisson Gap scheduling. J. Biomol. NMR 2012, 52, 315–327. 10.1007/s10858-012-9611-z. 22331404 PMC3321367

[ref36] Delaglio F.; Grzesiek S.; Vuister G. W.; Zhu G.; Pfeifer J.; Bax A. NMRPipe: a multidimensional spectral processing system based on UNIX pipes. J. Biomol. NMR 1995, 6, 277–293. 10.1007/BF00197809. 8520220

[ref37] Vranken W. F.; Boucher W.; Stevens T. J.; Fogh R. H.; Pajon A.; Llinas P.; Ulrich E. L.; Markley J. L.; Ionides J.; Laue E. D. The CCPN data model for NMR spectroscopy: Development of a software pipeline. Proteins 2005, 59, 687–696. 10.1002/prot.20449. 15815974

[ref38] Helmus J. J.; Jaroniec C. P. Nmrglue: an open source Python package for the analysis of multidimensional NMR data. J. Biomol. NMR 2013, 55, 355–367. 10.1007/s10858-013-9718-x. 23456039 PMC3636164

[ref39] Zhu G.; Xia Y.; Nicholson L. K.; Sze K. H. Protein Dynamics Measurements by TROSY-Based NMR Experiments. J. Magn. Reson. 2000, 143, 423–426. 10.1006/jmre.2000.2022. 10729271

[ref40] Kay L. E.; Torchia D. A.; Bax A. Backbone dynamics of proteins as studied by 15N inverse detected heteronuclear NMR spectroscopy: application to staphylococcal nuclease. Biochemistry 1989, 28, 8972–8979. 10.1021/bi00449a003. 2690953

[ref41] Lakomek N.-A.; Kaufman J. D.; Stahl S. J.; Louis J. M.; Grishaev A.; Wingfield P. T.; Bax A. Internal Dynamics of the Homotrimeric HIV-1 Viral Coat Protein gp41 on Multiple Time Scales. Angew. Chem., Int. Ed. 2013, 52, 3911–3915. 10.1002/anie.201207266. PMC3610801 23450638

[ref42] Lee D.; Hilty C.; Wider G.; Wuthrich K. Effective rotational correlation times of proteins from NMR relaxation interference. J. Magn. Reson. 2006, 178, 72–76. 10.1016/j.jmr.2005.08.014. 16188473

[ref43] d’Auvergne E. J.; Gooley P. R. Optimisation of NMR dynamic models I. Minimisation algorithms and their performance within the model-free and Brownian rotational diffusion spaces. J. Biomol. NMR 2008, 40, 107–119. 10.1007/s10858-007-9214-2. 18085410 PMC2758376

[ref44] Press W. H.; Teukolsky S. A.; Vetterling W. T.; Flannery B. P. Numerical Recipes: The Art of Scientific Computing, 3rd ed.; Cambridge University Press, 2007.

[ref45] Halle B.; Wennerström H. Interpretation of magnetic resonance data from water nuclei in heterogeneous systems. J. Chem. Phys. 1981, 75, 1928–1943. 10.1063/1.442218.

[ref46] Lipari G.; Szabo A. Model-Free Approach to the Interpretation of Nuclear Magnetic-Resonance Relaxation in Macromolecules. 1. Theory and Range of Validity. J. Am. Chem. Soc. 1982, 104, 4546–4559. 10.1021/ja00381a009.

[ref47] Clore G.; Szabo A.; Bax A.; Kay L.; Driscoll P.; Gronenborn A. Deviations From the Simple 2-Parameter Model-Free Approach to the Interpretation of N-15 Nuclear Magnetic-Relaxation of Proteins. J. Am. Chem. Soc. 1990, 112, 4989–4991. 10.1021/ja00168a070.

[ref48] Halle B. The physical basis of model-free analysis of NMR relaxation data from proteins and complex fluids. J. Chem. Phys. 2009, 131, 224507 10.1063/1.3269991. 20001057

[ref49] d’Auvergne E. J.; Gooley P. R. Set theory formulation of the model-free problem and the diffusion seeded model-free paradigm. Mol. BioSyst. 2007, 3, 483–494. 10.1039/b702202f. 17579774

[ref50] Walker O.; Varadan R.; Fushman D. Efficient and accurate determination of the overall rotational diffusion tensor of a molecule from 15N relaxation data using computer program ROTDIF. J. Magn. Reson. 2004, 168, 336–345. 10.1016/j.jmr.2004.03.019. 15140445

[ref51] Yao L.; Grishaev A.; Cornilescu G.; Bax A. Site-Specific Backbone Amide 15N Chemical Shift Anisotropy Tensors in a Small Protein from Liquid Crystal and Cross-Correlated Relaxation Measurements. J. Am. Chem. Soc. 2010, 132, 4295–4309. 10.1021/ja910186u. 20199098 PMC2847892

[ref52] Fushman D.; Tjandra N.; Cowburn D. Direct Measurement of 15N Chemical Shift Anisotropy in Solution. J. Am. Chem. Soc. 1998, 120, 10947–10952. 10.1021/ja981686m.

[ref53] Kroenke C.; Rance M.; Palmer A. Variability of the N-15 chemical shift anisotropy in *Escherichia coli* ribonuclease H in solution. J. Am. Chem. Soc. 1999, 121, 10119–10125. 10.1021/ja9909273.

[ref54] Ghose R.; Fushman D.; Cowburn D. Determination of the Rotational Diffusion Tensor of Macromolecules in Solution from NMR Relaxation Data with a Combination of Exact and Approximate Methods—Application to the Determination of Interdomain Orientation in Multidomain Proteins. J. Magn. Reson. 2001, 149, 204–217. 10.1006/jmre.2001.2295. 11318619

[ref55] García de la Torre J.; Huertas M. L.; Carrasco B. HYDRONMR: Prediction of NMR Relaxation of Globular Proteins from Atomic-Level Structures and Hydrodynamic Calculations. J. Magn. Reson. 2000, 147, 138–146. 10.1006/jmre.2000.2170. 11042057

[ref56] Bernadó P.; de la Torre J. G.; Pons M. Interpretation of 15N NMR relaxation data of globular proteins using hydrodynamic calculations with HYDRONMR. J. Biomol. NMR 2002, 23, 139–150. 10.1023/A:1016359412284. 12153039

[ref57] Halle B.; Davidovic M. Biomolecular hydration: From water dynamics to hydrodynamics. Proc. Natl. Acad. Sci. U.S.A. 2003, 100, 12135–12140. 10.1073/pnas.2033320100. 14528004 PMC218725

[ref58] Farrow N. A.; Zhang O.; SZABO A.; Torchia D. A.; Kay L. E. Spectral density function mapping using 15N relaxation data exclusively. J. Biomol. NMR 1995, 6, 153–162. 10.1007/BF00211779. 8589604

[ref59] Palmer A. G.; Kroenke C. D.; Loria J. P. [10] Nuclear Magnetic Resonance Methods for Quantifying Microsecond-to-Millisecond Motions in Biological Macromolecules. Nuclear Magnetic Resonance of Biological Macromolecules, Methods in Enzymology; Elsevier Inc., 2001; Vol. 339, pp 204–238. 10.1016/s0076-6879(01)39315-1 11462813

[ref60] Williams F. P.; Milbradt A. G.; Embrey K. J.; Bobby R. Segmental Isotope Labelling of an Individual Bromodomain of a Tandem Domain BRD4 Using Sortase A. PLoS One 2016, 11, e0154607 10.1371/journal.pone.0154607. 27128490 PMC4851411

[ref61] Patel K.; Solomon P. D.; Walshe J. L.; Ford D. J.; Payne R. J.; Wilkinson-White L.; Low J. K. K.; Mackay J. P. BET-Family Bromodomains Can Recognize Diacetylated Sequences from Transcription Factors Using a Conserved Mechanism. Biochemistry 2021, 60, 648–662. 10.1021/acs.biochem.0c00816. 33620209

[ref62] Wu S.-Y.; Chiang C.-M. The Double Bromodomain-containing Chromatin Adaptor Brd4 and Transcriptional Regulation. J. Biol. Chem. 2007, 282, 13141–13145. 10.1074/jbc.R700001200. 17329240

[ref63] Nakamura Y.; Umehara T.; Nakano K.; Jang M. K.; Shirouzu M.; Morita S.; Uda-Tochio H.; Hamana H.; Terada T.; Adachi N.; Matsumoto T.; Tanaka A.; Horikoshi M.; Ozato K.; Padmanabhan B.; Yokoyama S. Crystal Structure of the Human BRD2 Bromodomain. J. Biol. Chem. 2007, 282, 4193–4201. 10.1074/jbc.M605971200. 17148447

[ref64] Filippakopoulos P.; Picaud S.; Mangos M.; Keates T.; Lambert J.-P.; Barsyte-Lovejoy D.; Felletar I.; Volkmer R.; Müller S.; Pawson T.; Gingras A.-C.; Arrowsmith C. H.; Knapp S. Histone recognition and large-scale structural analysis of the human bromodomain family. Cell 2012, 149, 214–231. 10.1016/j.cell.2012.02.013. 22464331 PMC3326523

[ref65] Vollmuth F.; Blankenfeldt W.; Geyer M. Structures of the dual bromodomains of the P-TEFb-activating protein Brd4 at atomic resolution. J. Biol. Chem. 2009, 284, 36547–36556. 10.1074/jbc.M109.033712. 19828451 PMC2794770

[ref66] Morinière J.; Rousseaux S.; Steuerwald U.; Soler-López M.; Curtet S.; Vitte A.-L.; Govin J.; Gaucher J.; Sadoul K.; Hart D. J.; Krijgsveld J.; Khochbin S.; Müller C. W.; Petosa C. Cooperative binding of two acetylation marks on a histone tail by a single bromodomain. Nature 2009, 461, 664–668. 10.1038/nature08397. 19794495

[ref67] Umehara T.; Nakamura Y.; Jang M. K.; Nakano K.; Tanaka A.; Ozato K.; Padmanabhan B.; Yokoyama S. Structural Basis for Acetylated Histone H4 Recognition by the Human BRD2 Bromodomain. J. Biol. Chem. 2010, 285, 7610–7618. 10.1074/jbc.M109.062422. 20048151 PMC2844208

[ref68] Patel K.; Walport L. J.; Walshe J. L.; Solomon P. D.; Low J. K. K.; Tran D. H.; Mouradian K. S.; Silva A. P. G.; Wilkinson-White L.; Norman A.; Franck C.; Matthews J. M.; Guss J. M.; Payne R. J.; Passioura T.; Suga H.; Mackay J. P. Cyclic peptides can engage a single binding pocket through highly divergent modes. Proc. Natl. Acad. Sci. U.S.A. 2020, 117, 26728–26738. 10.1073/pnas.2003086117. 33046654 PMC7604503

[ref69] Mulder F. A. A.; Bouakaz L.; Lundell A.; Venkataramana M.; Liljas A.; Akke M.; Sanyal S. Conformation and dynamics of ribosomal stalk protein L12 in solution and on the ribosome. Biochemistry 2004, 43, 5930–5936. 10.1021/bi0495331. 15147176

[ref70] Walsh J. D.; Meier K.; Ishima R.; Gronenborn A. M. NMR Studies on Domain Diffusion and Alignment in Modular GB1 Repeats. Biophys. J. 2010, 99, 2636–2646. 10.1016/j.bpj.2010.08.036. 20959105 PMC2955504

[ref71] Gill M. L.; Palmer A. G. III Local Isotropic Diffusion Approximation for Coupled Internal and Overall Molecular Motions in NMR Spin Relaxation. J. Phys. Chem. B 2014, 118, 11120–11128. 10.1021/jp506580c. 25167331 PMC4174990

[ref72] Goodman J. L.; Pagel M. D.; Stone M. J. Relationships between protein structure and dynamics from a database of NMR-derived backbone order parameters. J. Mol. Biol. 2000, 295, 963–978. 10.1006/jmbi.1999.3419. 10656804

[ref73] Rodríguez Y.; Navarro G. G.; Osman R.; Zhou M.-M. In silico design and molecular basis for the selectivity of Olinone toward the first over the second bromodomain of BRD4. Proteins 2020, 88, 414–430. 10.1002/prot.25818. 31587361 PMC6982606

[ref74] Malvezzi F.; Stubbs C. J.; Jowitt T. A.; Dale I. L.; Guo X.; DeGnore J. P.; Degliesposti G.; Skehel J. M.; Bannister A. J.; McAlister M. S. Phosphorylation-dependent BRD4 dimerization and implications for therapeutic inhibition of BET family proteins. Commun. Biol 2021, 4, 1273 10.1038/s42003-021-02750-6. 34754068 PMC8578508

[ref75] Lori L.; Pasquo A.; Lori C.; Petrosino M.; Chiaraluce R.; Tallant C.; Knapp S.; Consalvi V. Effect of BET Missense Mutations on Bromodomain Function, Inhibitor Binding and Stability. PLoS One 2016, 11, e0159180 10.1371/journal.pone.0159180. 27403962 PMC4942050

