# Supplementary material for: Bromodomain Interactions with Acetylated Histone 4 Peptides in the BRD4 Tandem Domain: Effects on Domain Dynamics and Internal Flexibility
Source: Biochemistry. 2022 Oct 10;61(21):2303–18. doi: 10.1021/acs.biochem.2c00226 (PMC9631989; doi:10.1021/acs.biochem.2c00226)
Supplement: Supplementary file 1 — bi2c00226_si_001.pdf [file bi2c00226_si_001.pdf]

Supplementary Information

Bromodomain Interactions with Acetylated Histone 4 Peptides in the BRD4 Tandem Domain: Effects on Domain Dynamics and Internal Flexibility

Sven Wernersson,[1,‡] Romel Bobby,[2,4,‡] Liz Flavell,[3,5] Alexander G. Milbradt,[2] Geoffrey A. Holdgate,[2,6] Kevin J. Embrey,[2,7] and Mikael Akke[1]**

[1] Biophysical Chemistry, Center for Molecular Protein Science, Department of Chemistry, Lund University, SE-221 00 Lund, Sweden
[2] Mechanistic and Structural Biology, Discovery Sciences, BioPharmaceuticals R&D, AstraZeneca, Cambridge, UK
[3] Discovery Biology, Discovery Sciences, BioPharmaceuticals R&D, AstraZeneca, Cambridge Science Park, Cambridge, UK

Present address:
[4] Roche Pharma Research and Early Development, pRED Informatics, Roche Innovation Center Basel, F. Hoffmann-La Roche Ltd, Grenzacherstrasse 124, 4070 Basel, Switzerland
[5] Peak Proteins Ltd., Birchwood House, Larkwood Way, Tytherington Business Park, Macclesfield, SK10 2XR, UK
[6] High-throughput Screening, Discovery Sciences, BioPharmaceuticals R&D, AstraZeneca, Alderley Park, UK
[7] New Modalities Product Development, Pharmaceutical Technology & Development, Operations, AstraZeneca, Macclesfield, UK

* Corresponding Authors: Kevin J. Embrey, kevin.embrey@astrazeneca.com; Mikael Akke, mikael.akke@bpc.lu.se

Table S1. Trimmed averages of all NMR relaxation data.

14.1 T data

apo constructs	R_1 (s^{-1})[a]	R_2 (s^{-1})[b]	NOE[c]
BD1 (isolated)	1.33	13	0.79
BD2 (isolated)	1.09	15	0.80
BD1 (tandem)	0.97	22	0.81
BD2 (tandem)	0.94	26	0.79
H4Kac-4 bound constructs			
BD1 (isolated)	1.13	16	0.81
BD2 (isolated)	0.94	17	0.79
BD1 (tandem)	0.77	25	0.81
BD2 (tandem)	0.79	30	0.81

18.8 T data

apo constructs	R_1 (s^{-1})[a]	R_2 (s^{-1})[b]	NOE[c]	η_{xy} (s^{-1})[d]
BD1 (isolated)	0.97	15	0.83	11
BD2 (isolated)	0.80	18	0.84	14
BD1 (tandem)	0.60	26	0.87	22
BD2 (tandem)	0.67	32	0.86	25
H4Kac-4 bound constructs				
BD1 (isolated)	0.85	18	0.83	N.D.[e]
BD2 (isolated)	0.68	22	0.85	17
BD1 (tandem)	0.55	30	0.86	26
BD2 (tandem)	0.55	37	0.85	30

[a]The standard error of the mean (SEM) is ≤ 0.005 in all cases. [b]SEM ≤ 0.1 in all cases. [c]SEM ≤ 0.01 in all cases. [d]SEM ≤ 0.3 in all cases. [e]N.D. = No data.

Table S2: Diffusion tensor parameters determined with the relax software suite.[a]

apo constructs			
Construct	D_{iso} (10^7 s^{-1})	$D_{\|\|}/D_{\perp}$	τ_c (ns)
BD1 (isolated)	1.90	1.41	8.8
BD2 (isolated)	1.62	1.30	10.3
BD1 (tandem)	1.23	1.80	13.5
BD2 (tandem)	1.06	1.12	15.7
H4Kac-4 bound constructs			
Construct	D_{iso} (10^7 s^{-1})	$D_{\|\|}/D_{\perp}$	τ_c (ns)
BD1 (isolated)	1.36	1.79	12.3
BD2 (isolated)	1.44	0.56	11.6
BD1 (tandem)	1.04	1.67	16.1
BD2 (tandem)	1.13	1.77	14.7

[a] D_{iso} is the trace of the diffusion tensor, ($D_{||}/D_{\perp}$) is the anisotropy of the diffusion tensor, τ_c is the rotational diffusion correlation time. See the main text for definitions.

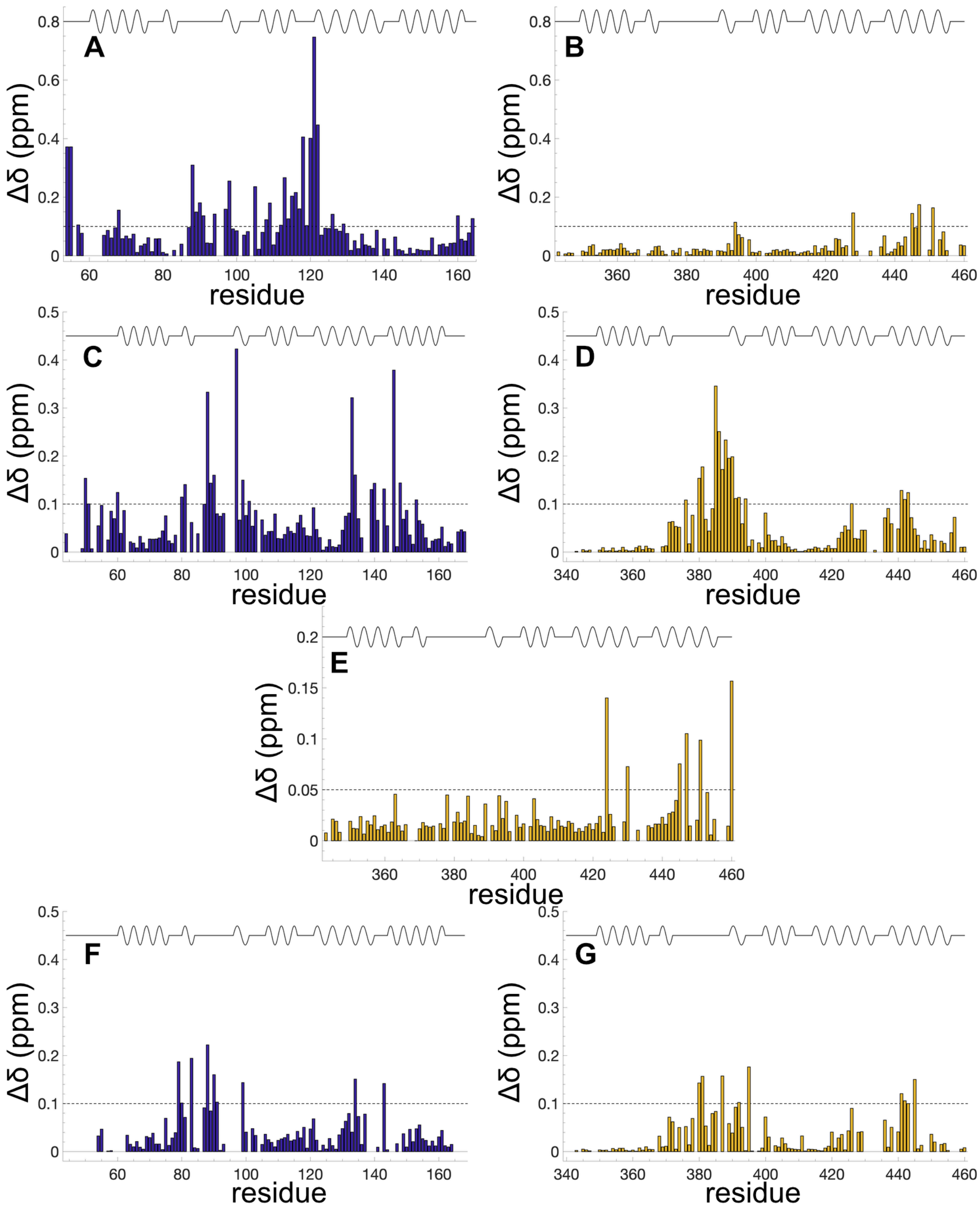

Figure S1. Chemical shift differences between different states or constructs of BRD4. (A) Differences between: (A) tandem – isolated BD1; (B) tandem – isolated BD2; (C) H4Kac4-bound – apo isolated BD1; (D) H4Kac4-bound – apo isolated BD2; (E) concentration-dependent chemical shift changes for isolated BD2: 526 μM BD2 – 120 μM BD2; (F) H4Kac4-bound – apo tandem BD1; (G) H4Kac4-bound – apo tandem BD2. Chemical shift changes greater than the

values indicated by the dashed lines are highlighted on the structures in Figs. 2 and 3 of the main text.

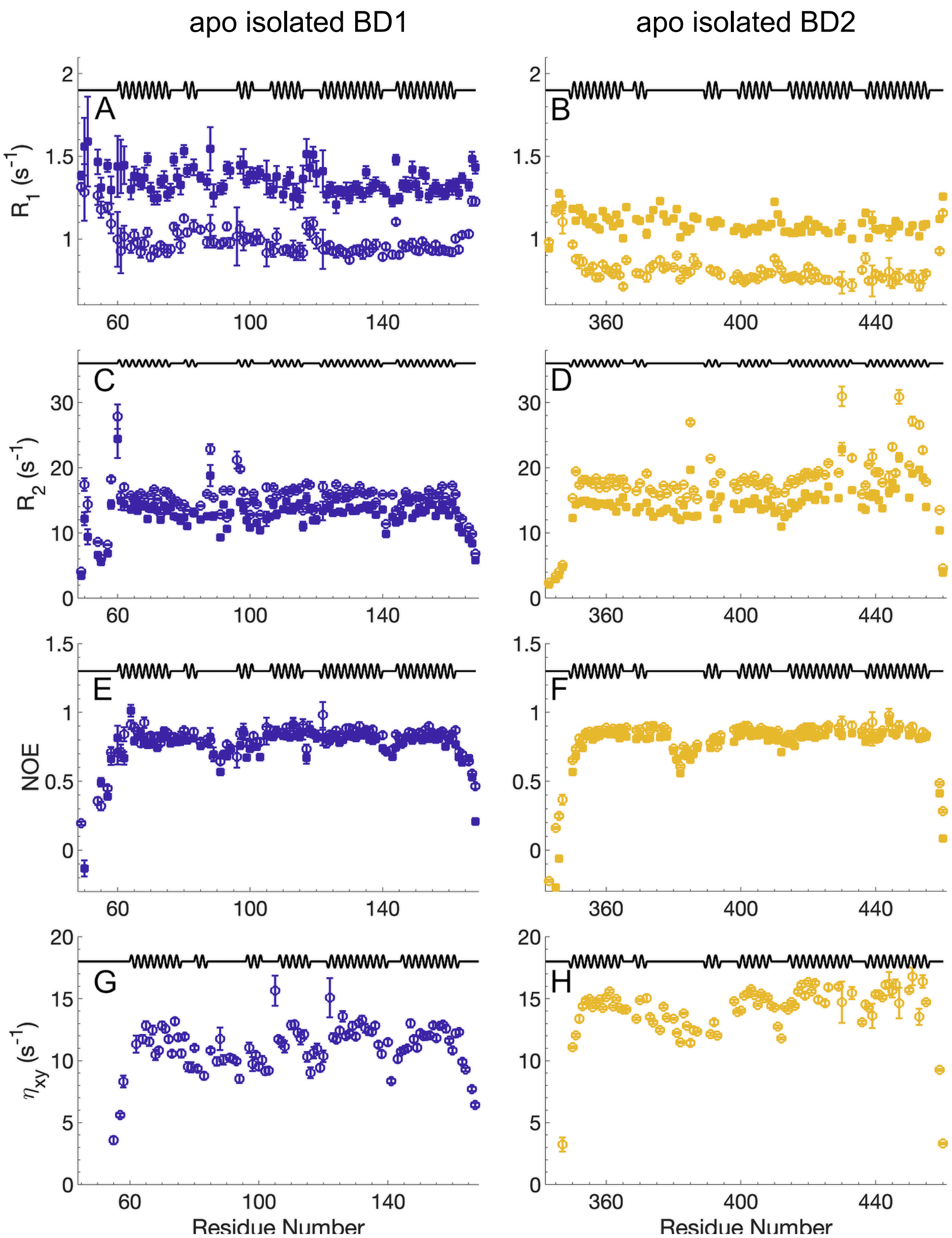

Figure S2. Relaxation data of apo, isolated BRD4 bromodomains at 14.1 T (filled squares) and 18.8 T (open circles). (A, B) R_1, (C, D) R_2, (E, F) $\{^1H\}$-^{15}N *NOE*, (G, H) η_{xy}. Left-hand column (A, C, E, G) apo isolated BD1. Right-hand column (B, D, F, H) apo isolated BD2.

The black line at the top of each panel shows the secondary structure, with α-helices represented as waves and intervening segments (loops and linker) as straight lines.

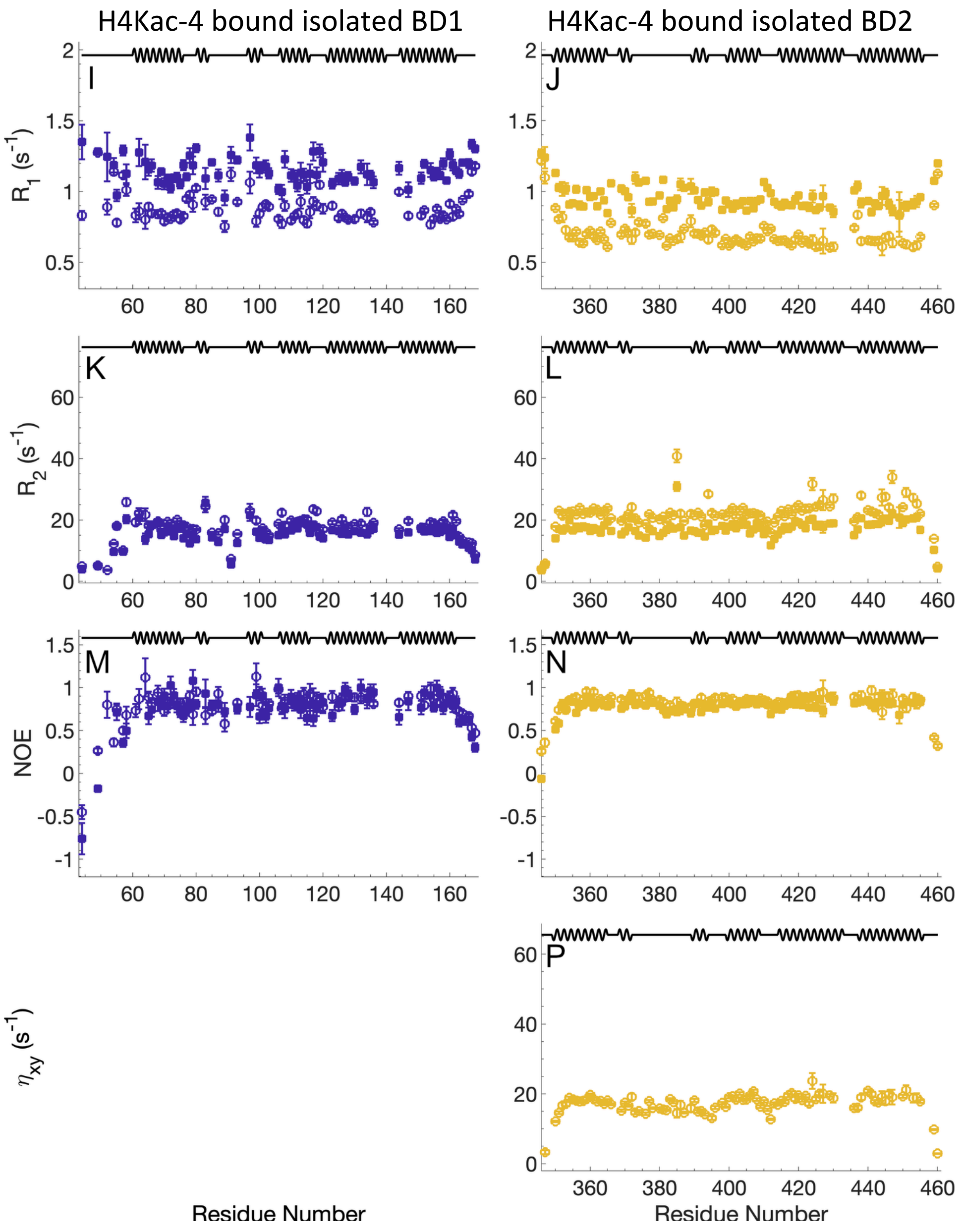

Figure S2, continued. Relaxation data of H4Kac-4 bound, isolated BRD4 bromodomains at 14.1 T (filled squares) and 18.8 T (open circles). (I, J) R_1, (K, L) R_2, (M, N) {^{1}H}-^{15}N *NOE*, (P) η_{xy}. Left-hand column (I, K, M) H4Kac-4 bound isolated BD1. Right-hand column (J, L, N, P) H4Kac-4 bound isolated BD2. The black line at the top of each panel shows the secondary structure, with α-helices represented as waves and intervening segments (loops and linker) as straight lines. Panel O is intentionally omitted because the corresponding data are not available.

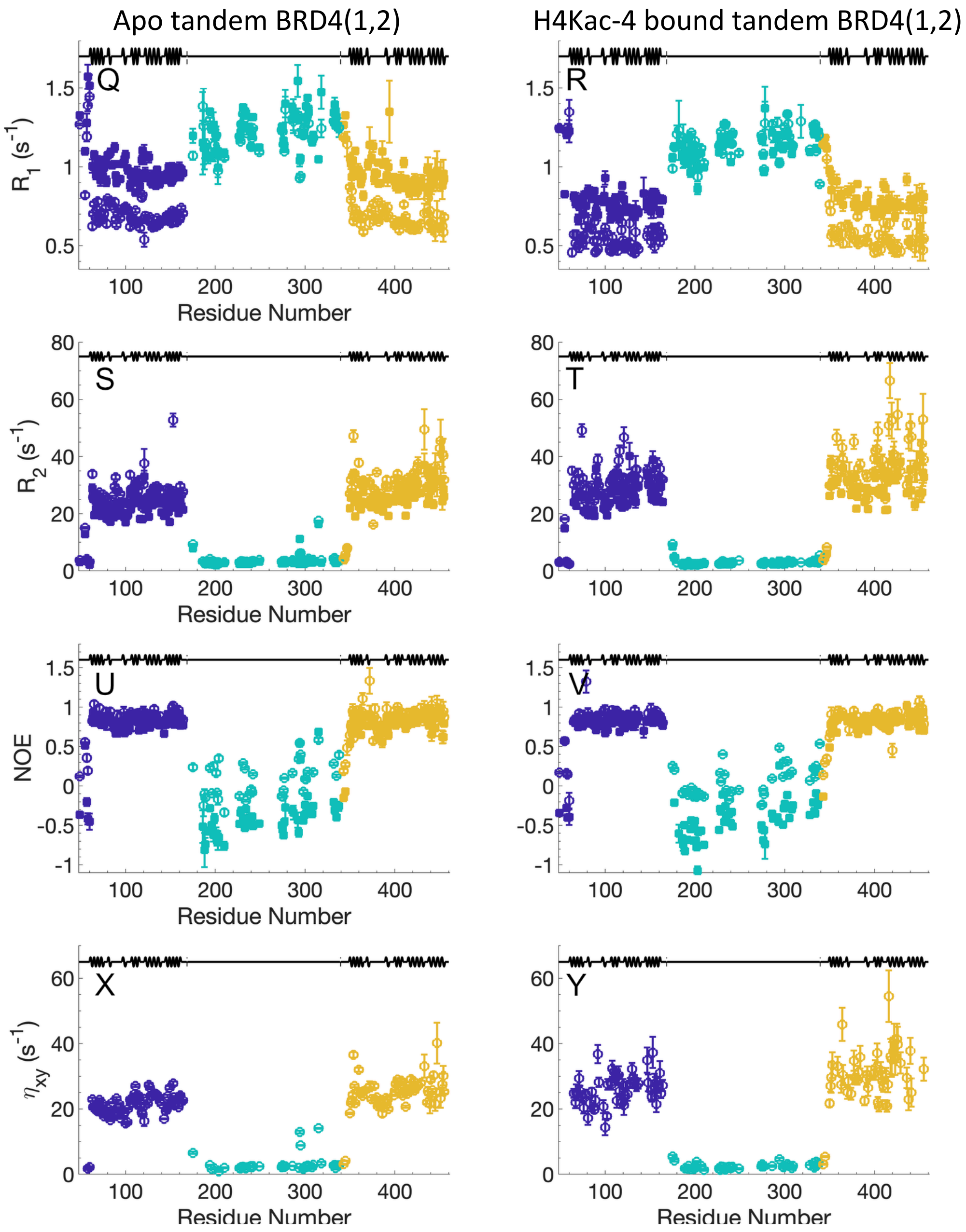

Figure S2, continued. Relaxation data of tandem BRD4(1,2) at 14.1 T (filled squares) and 18.8 T (open circles). (Q, R) R_1, (S, T) R_2, (U, V) {^{1}H}-^{15}N *NOE*, (X, Y) η_{xy}. Left-hand column (Q, S, U, X) Apo tandem BRD4(1,2). Right-hand column (R, T, V, Y) H4Kac-4 bound tandem BRD4(1,2). The black line at the top of each panel shows the secondary structure, with α-helices represented as waves and intervening segments (loops and linker) as straight lines.

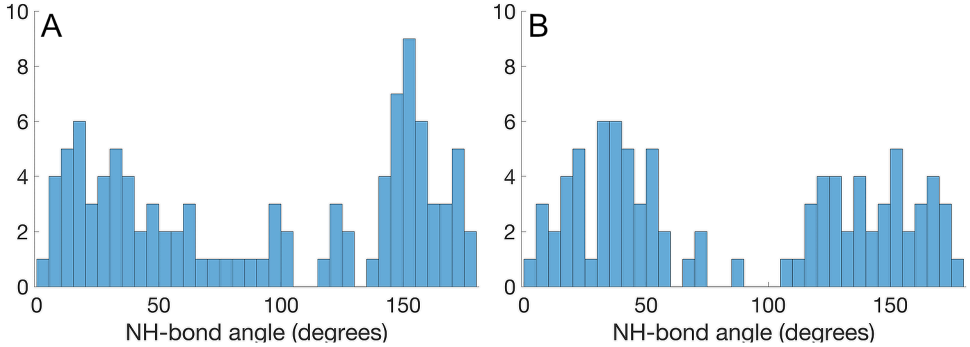

Figure S3. Histograms of N-H bond vector angles relative to the unique axis of the diffusion tensors calculated using rotdif. Each histogram contains 36 bins with a width of 5°. (A) Apo, isolated BD1, (B) Apo, isolated BD2.

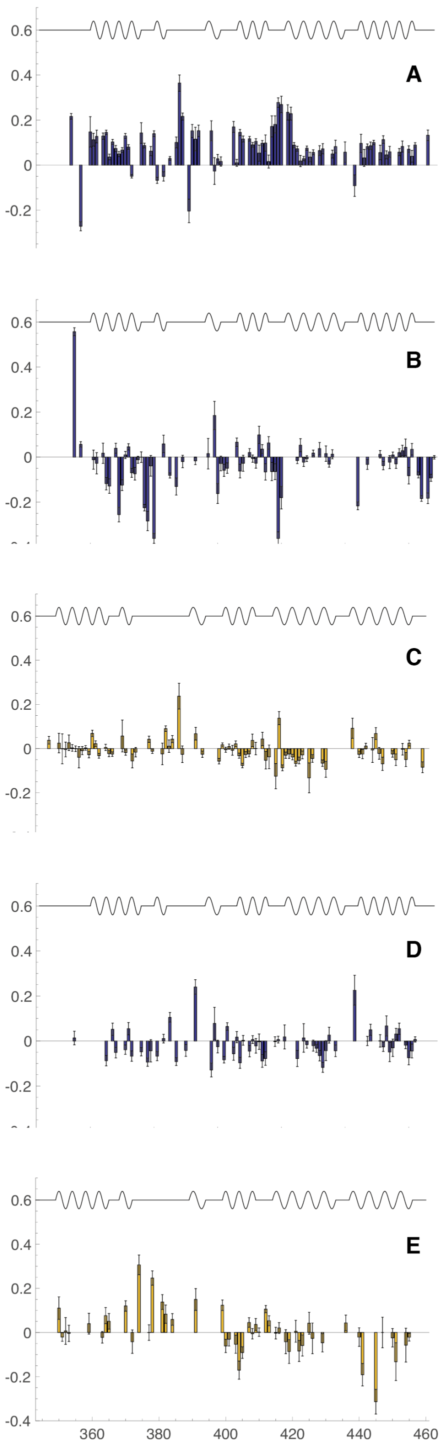

Figure S4. Difference in S^2 between different states of BRD4 bromodomains (corresponding to Figures 5 and 6 of the main text). (A-C) isolated domains: (A) $\Delta S^2 = S^2$(apo BD1) – S^2(apo BD2) (cf. Fig 5A of the main text); (B) $\Delta S^2 = S^2$(HKac-4 bound BD1) – S^2(apo BD1); (C) $\Delta S^2 = S^2$(HKac-4

bound BD2) – S^2(apo BD2). (D, E) tandem constructs: (E) ΔS^2 = S^2(HKac-4 bound BD1) – S^2(apo BD1); (E) ΔS^2 = S^2(HKac-4 bound BD2) – S^2(apo BD2).
